# Protocol for a quasi experimental mixed method study on impact of intervention for improving Infant and Young Child Feeding (IYCF) practices in tribal block of Palghar District, Maharashtra, India through involvement of frontline workers

**DOI:** 10.1371/journal.pone.0353241

**Published:** 2026-07-15

**Authors:** Suchitra Surve, Shraddha Kyatam, Shahina Begum, Bhavya M. K, Santosh Chaudhari, Sagar Patil, Shivshankar Timmanpyati, Purabi Mahajan, Ragini Kulkarni

**Affiliations:** 1 Scientist D, Department of Child Health Research, ICMR-NIRRCH, Mumbai, Maharashtra, India; 2 Project Research Scientist-I (Non-medical), Department of Operational and Implementation Research, MRHRU Dahanu, ICMR-NIRRCH, Mumbai, Maharashtra, India; 3 Scientist F, Department of Biostatistics and Socio Behavioral Research, ICMR-NIRRCH, Mumbai, Maharashtra, India; 4 Scientist B, Department of Operational and Implementation Research, ICMR-NIRRCH, Mumbai, Maharashtra, India; 5 District Health Officer, Office of the District Health Officer, District Headquarters, Palghar, Maharashtra, India; 6 District RCH Officer, Office of the District Health Officer, District Headquarters, Palghar, Maharashtra, India; 7 Chief Clinical Dietician, Tata Memorial Hospital, Mumbai, Maharashtra, India; 8 Senior Clinical Dietician, Tata Memorial Hospital, Mumbai, Maharashtra, India; 9 Scientist F, Department of Operational and Implementation Research, Nodal Officer- MRHRU Dahanu, ICMR-NIRRCH, Mumbai, Maharashtra, India; All India Institute of Medical Sciences - Raipur, INDIA

## Abstract

**Introduction:**

Infant and Young Child Feeding (IYCF) is crucial for child growth, especially in the first two years. Despite various government initiatives, nearly 35.5% of under-five children present with stunted growth in India as per NFHS-5 survey. Moreover, tribal areas like Palghar in India are most affected due to limited access to healthcare, poverty and knowledge gaps. While frontline workers such as Anganwadi Workers (AWWs) play a key role in IYCF promotion in community; gaps in their knowledge, counselling strategies and contextual barriers hamper the desired outcome. The study aims to assess the knowledge, attitude, and practices (KAP) of frontline workers and mothers on IYCF; and to develop and evaluate a context specific intervention package to improve IYCF practices for better health outcomes among young children.

**Methods:**

A quasi-experimental mixed-methods study will be conducted over two years in three phases (baseline, intervention and post intervention) in the Ganjad PHC of Palghar District, Maharashtra. After qualitative assessment of frontline workers, a total of 460 mother-infant (6–12 months) dyads will be enrolled in baseline and post intervention through cluster sampling across 44 Anganwadi Centres. Baseline and endline IYCF practices will be collected using a structured questionnaire along with anthropometric assessment The intervention will include capacity building of frontline workers and mothers on IYCF Practices through Socio Behavioral Communication Change approach, preparation of pictorial booklets and charts, provision of hot cooked CF servings at Anganwadi Centers along with Iron, Calcium and Vitamin D supplementation. The quantitative data will be analyzed using descriptive and inferential statistics, and qualitative data thematically.

**Discussion:**

This study proposes a community-based intervention to improve IYCF practices among tribal populations in Palghar District, Maharashtra. It integrates behaviour change communication with hot cooked meal provision at Anganwadi Centres through frontline workers. The approach emphasizes cultural relevance and sustainability, with potential to inform scalable strategies and policy within ICDS.

**Clinical trial registration:**

CTRI/2024/06/068427 Registered on 06th June, 2024.

## Introduction

Undernutrition remains a major contributor of child mortality, accounting for nearly 50% of child deaths. It mainly prevails during the initial two years of life which is a crucial window for child growth and development and when nutritional requirements are at the peak [1].

Infant and Young Child Feeding (IYCF) practices comprise a continuum of appropriate feeding of newborn and children under two years of age, i.e., exclusive breastfeeding, initiation of complementary feeding (CF) and gradual transition to family diet [2]. The commencement of CF entails gradual introduction of diverse foods until the child adapts to eating from the family pot [3,4]. This phase is particularly crucial as it is marked by increasing nutritional needs for optimal growth and development.

Inappropriate CF practices such as delayed initiation, inadequate nutritional content, low nutrient density, feeding in small amounts, inadequate meal frequency and food restrictions are predominantly associated with growth faltering, malnutrition and micronutrient deficiencies. It may also lead to increased risk of infections and mortality during the first two years of life, [5–7]. Sub-optimal child feeding practices not only affect physical health of a child, but also impair cognitive and social development leading to poor academic performance [2].

WHO has identified eight main core indicators of IYCF practices namely- early initiation of breastfeeding, exclusive breastfeeding for six months, continued breastfeeding till completion of first year, Introduction of solid, semi-solid or soft foods, Minimum Dietary Diversity (MDD), Minimum Meal Frequency (MMF), Minimum Acceptable Diet (MAD) and Consumption of iron rich or iron-fortified foods [4]. However, IYCF practices are often influenced by interplay of various factors including poor hygiene, traditional feeding customs and local or regional food availability. Moreover, these practices are often suboptimal in tribal regions due to poor nutrition, cultural beliefs, geographical disparities, poor socio-economic status and inadequate health facilities [8]. This leads to undernutrition in tribal children as a result of inadequate nutritional intake and subsequent higher rates of morbidity [9].

Several studies done so far in Indian population have shown that inappropriate IYCF practices are higher among tribal population, [10–12]. However, very few studies have evaluated impact of nutrition education on variety, quantity and consistency of CF. There is scarcity of data on assessing improvement in IYCF core indicators through community-based intervention, [13,14]. Therefore, it is critical to identify approaches to improve IYCF practices to achieve optimal nutrition.

While frontline workers such as Accredited Social Health Activists (ASHAs), Anganwadi Workers (AWWs), and Auxiliary Nurse Midwives (ANMs) play a pivotal role in promoting behaviour change and providing nutrition education at the community level, their potential is underutilized due to lack of refresher trainings, context specific socio-behavioral communication change strategies.

Therefore, this study aims to improve IYCF practices in a tribal block of Palghar district through involvement of frontline workers for promoting better health outcomes among infants and young children.

## Materials and methods

### Study design

A quasi-experimental mixed-methods study will be conducted using pre and post-intervention assessments among mothers of infants in a tribal block of Palghar, Maharashtra. Changes in knowledge, attitudes and practices (KAP) will be assessed quantitatively and complemented with qualitative insights to evaluate the effectiveness of a frontline worker followed by IYCF intervention. The TREND checklist for quai experimental study (S1 File) and COREQ (S2 File) checklist for qualitative research will be followed to ensure comprehensive and transparent reporting.

### Study setting

The study will be conducted in Primary Health Centre (PHC) area Ganjad which is located in the tribal region of Palghar District, Maharashtra, India. This area is predominantly inhabited by tribal communities (Kathodi, Katkari, Kokana, Koli Mahadeo, Koli Malhar, Warli, Vanjari, Thakar, Thakur, Dubala, Dhor Koli and Tokre Koli) and is characterized by limited access to healthcare services due to geographical disparities. The PHC serves as the primary healthcare facility for the surrounding villages (Table 1) and will act as the central site for implementing and monitoring the study activities.

**Table 1:** List of Villages with Number of Padas, Anganwadi Centers (AWCs), and Anganwadi Workers (AWWs) under Ganjad PHC.

Village Name	Number of Padas	Number of AWCs	Number of AWWs
Ganjad	19	19	19
Raitali	7	7	7
Chari	11	11	11
Jamshet	15	15	15
Ambesari	12	12	12
Nagzari	13	13	13
**Total**	**77**	**77**	**77**

Abbreviations: AWC – Anganwadi Center, AWW- Anganwadi Worker, PHC- Primary Health Center.

There are six subcentres in Ganjad PHC: Ganjad, Raitali, Chari, Jamshet, Ambesari and Nagzari. The subcentre wise list of padas is mentioned in Table 1. Each pada is served by one Anganwadi Centre (AWC), resulting in 77 AWCs across the six villages. AWC is a community-based facility in India for children aged 0–6 years and pregnant or lactating women established under India’s Integrated Child Development Services (ICDS) programme. It provides supplementary nutrition, immunization, health check-ups, and preschool education to children less than six years of age, as well as aid to pregnant and breastfeeding mothers. Each center is managed by a trained Anganwadi Worker (AWW) and serves as an important link between the community and the health system, especially in rural and tribal areas, helping to reduce malnutrition, improve maternal and child health and support early childhood development. Correspondingly, there are 77 Anganwadi Workers (AWWs), with one assigned to each AWC and ten AWC are supervised by one AWC supervisor. Furthermore, Accredited Social Health Activists (ASHAs), a crucial part in rural health settings in India, are female community health workers, playing a key role in health awareness and community mobilization.

### Sample size

Considering 48.3% mothers who timely initiated complementary feeding at six months as per findings of previous study conducted by authors in Palghar District [15] and anticipating 10% improvement post intervention with 5% level of significance and 80% power, nearly 410 children will be required. Further, considering 10% non-response rate, 456 children will be enrolled. Therefore, an independent sample of 460 children will be selected in pre intervention and post intervention survey (10–11 children per AWC). The sample size formula is as follows;


$$\begin{array}{c} 𝐧=\frac{{\left[{𝐙}_{1-\alpha /2}\cdot \sqrt{2𝐏\left(1-𝐏\right)}+{𝐙}_{1-\beta }\cdot \sqrt{\{{𝐏}_{1}\left(1-{𝐏}_{1}\right)+{𝐏}_{2}\left(1-{𝐏}_{2}\right)\}}\right]}^{2}}{{\left({𝐏}_{1}-{𝐏}_{2}\right)}^{2}} \end{array}$$

**Where, P = (P**_**1**_ **+ P**_**2**_**) / 2; P**_**1**_ **= 48.3%, P**_**2**_ **= 58.3%.**

### Study population

The study population will comprise infants aged 6–12 months attending Anganwadi Centres (AWCs) along with their mothers, who will be enrolled as participants in the study. Infants with chronic illnesses such as congenital heart disease, diabetes, or cancer, as well as those diagnosed with Severe Acute Malnutrition (SAM), Moderate Acute Malnutrition (MAM), or congenital anomalies such as cleft lip and/or cleft palate, will be excluded. Infants whose mothers do not provide informed consent will also be excluded. In addition, frontline workers, including Anganwadi Workers (AWWs), Anganwadi Supervisors and Accredited Social Health Activists (ASHAs), will be included as study participants.

### Ethics statement

#### Institutional review board statement.

This study received ethical approval from the ICMR-NIRRCH Ethics Committee for Human Bodies on November 13^th^, 2023 (D/ICEC/Sci-225/238/2023)

#### Informed consent statement.

Written informed consent will be obtained from the parents of all participating children prior to their inclusion in the study. Considering that the participants are children from rural and tribal areas, they are considered a vulnerable population. Therefore, special care will be taken to ensure ethical standards are upheld. The parents will be provided with a Participant Information Sheet clearly outlining the purpose and objectives of the study in a language they understand. Participation will be entirely voluntary, and no form of coercion or undue influence will be used to encourage participation. For illiterate parents, they will be explained the PIS in the local language in the presence of witness, their thumb impression will be taken and signature of witness will be taken. The privacy and confidentiality of all participants will be strictly maintained throughout the study. Personal and health-related data will be anonymized and handled with care. All physical records will be stored securely under lock and key, while electronic data will be stored in password-protected systems accessible only to the Principal Investigator and authorized research staff.

### Study duration

**Fig 1:**
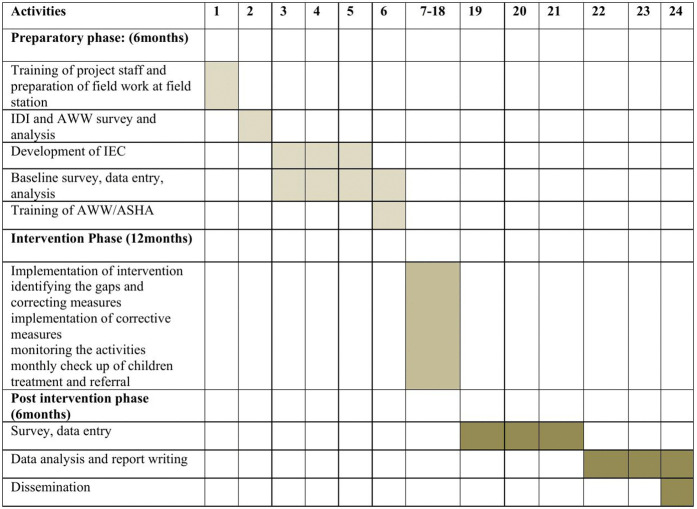
Gantt chart. Gantt chart illustrating the timeline of study phases and key activities over the two-year study period, including pre-intervention, intervention and post-intervention phases.

The study will be conducted over a total period of 24 months, including a 6-month preparatory phase, a 12-month intervention phase and a 6-month post-intervention phase.Activities will include baseline assessments, intervention implementation with monitoring, followed by endline survey, data analysis and dissemination, as outlined in Fig 1.

### Study framework

**Fig 2:**
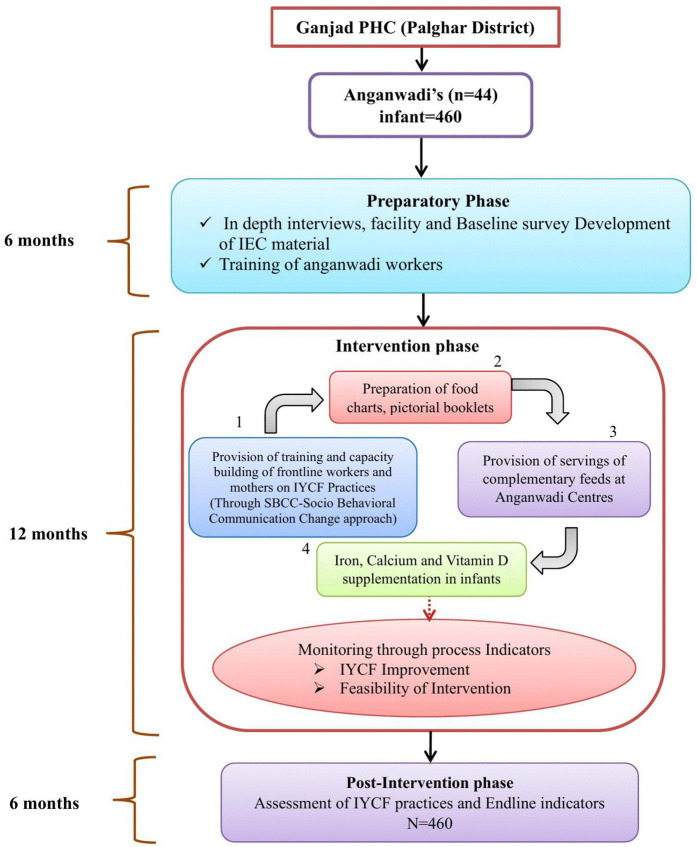
The study framework. The flowchart illustrates a phased intervention model at PHC-Ganjad involving 6 months preparatory activities, a 12-month intervention package, process monitoring and post intervention assessment across 44 Anganwadi’s covering 460 infants.

The study will be implemented over two years, in three phases, viz., Pre-intervention Phase, Intervention phase and post-intervention phase as depicted in study framework (Fig 2)

### Pre-intervention phase

#### 1. Preparatory activities.

During the pre-intervention phase, initially preparatory activities will include meetings with district health officials and training of staff recruited for the study. A comprehensive meeting with key district officials, including the Deputy Chief Executive Officer of the Women and Child Development Department, Palghar, and the CDPO of Dahanu block. This meeting will serve to provide a detailed overview of proposed study, outlining its objectives, implementation strategies and expected outcomes, thereby ensuring alignment and collaboration among all stakeholders to tackle malnutrition and promote optimal feeding practices.

#### 2. Data collection (qualitative and quantitative).

As part of preparatory activities of the study, the situational analysis will be done from February 2024 to September 2024, through in-depth interviews (IDIs) with health care providers and frontline workers to collect information about their knowledge and perceptions about IYCF practices. The number of IDIs will be determined by convenient sampling. IDIs will be planned with CDPO, Anganwadi Supervisors, AWWs and ASHA workers. (S3 File–3.1,3.2, 3.3, 3.4, 3.5 – IDI Guide)

As part of the baseline data collection, information about IYCF practices and child’s health status will be collected from 460 eligible mothers in a structured data collection form using the eight core indicators of IYCF practices. These include early initiation of breastfeeding, exclusive breastfeeding for the first six months, continued breastfeeding at one-year, timely introduction of solid, MDD, MMF, MAD, full forms and the consumption of iron-rich or iron-fortified foods (S4 File- Data Collection tool -Mothers and Children). The inclusion criteria for the study consist of infants aged between 6 to 12 months who visit AWCs along with their mothers who are also enrolled in the study. The infants with chronic illnesses such as congenital heart disease, diabetes, cancer, or those diagnosed with Severe Acute Malnutrition (SAM), Moderate Acute Malnutrition (MAM) and birth defects like cleft lip or cleft palate will be excluded. Furthermore, any participant whose mothers does not provide consent will be excluded from the study.

The beneficiaries will be approached at Anganwadi Centres as shown in study framework. (Fig 2).

Additionally, a baseline survey will be conducted with ASHA workers and AWWs. They will be randomly selected through the Ganjad Primary Health Centre (PHC) area.

#### 3. Preparation of intervention activities.

To develop an intervention package, the existing IEC (Information, Education and Communication) materials on complementary feeding practices available at AWC will be reviewed, and improved IEC will be developed. A range of IEC materials will include a flip chart, video messages; pictorial charts etc. to promote behaviour change and improve IYCF practices. These will include information on optimal IYCF practices such as importance of exclusive breastfeeding, dos and don’ts of complementary feeding, continued breastfeeding and emphasis on iron and calcium-rich foods. Additional visual materials will feature information on appropriate semi-solid and soft foods and key IYCF indicators such as MDD, MMF and MAD. Pictorial recipe booklets and cooking videos demonstrating easy, nutritious recipes using locally available ingredients will be developed for better understanding and recall. IEC material will be developed in consultation with an expert committee, comprising of Nutritionists, Paediatricians in accordance to IYCF Guidelines of National Health Mission (NHM) [2], Manual on Dietary Guidelines for India, ICMR- National Institute of Nutrition (NIN), [16–19] (S5 File- Complementary feeding guidelines).

Pre-intervention phase will also include capacity-building sessions for AWWs along with audio visual sessions for AWWs and ASHAs focused on IYCF concepts. These sessions will include demonstrations of recipes made from locally available ingredients and the use of amylase-rich flour, with a special focus on simple and nutritious recipes derived from Take Home Ration (THR) provided at the Anganwadi’s. Counselling sessions will also emphasize the importance of avoiding processed foods.

The intervention will be pretested at the end of the preparatory phase to assess feasibility, acceptability and effectiveness before full-scale implementation.

### Intervention phase

The intervention phase will be initiated in October 2024. Infants aged 6–12 months and children’s who regularly visit to the anganwadi Centers, along with their mothers will be included during intervention phase regardless of their inclusion in preintervention phase to ensure intervention to all infants in desired age group in the study area. whereas infants suffering from chronic illnesses such as congenital heart disease, birth defects, diabetes, cancer, or other severe health conditions, those already diagnosed with Severe Acute Malnutrition (SAM), Moderate Acute Malnutrition (MAM) or those who are not willing to participate will also be excluded during the intervention phase.

During the intervention phase, several structured activities will be introduced to promote optimal IYCF practices in a defined timeline with emphasis on following four domains of intervention package, i.e.,

- Provision of training and capacity building of frontline workers and mothers on IYCF Practices through SBCC (Socio Behavioral Communication Change approach)
- Preparation of food charts, pictorial booklets
- Provision of servings of complementary feeds at Anganwadi Centres
- Iron, Calcium and Vitamin D supplementation in infants

**Fig 3:**
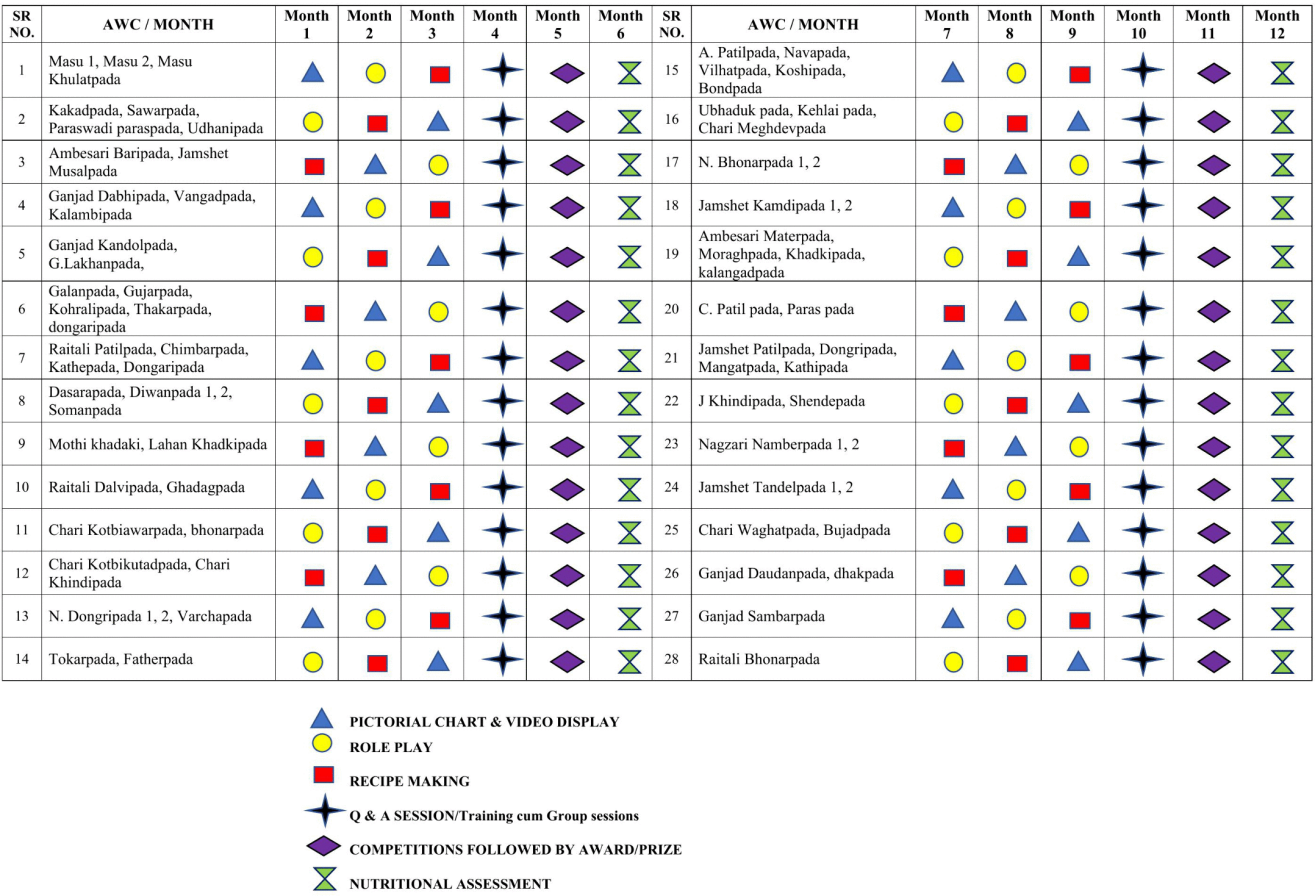
The intervention activities. The figure displays the monthly schedule of IYCF activities across Anganwadi Centers, using symbols to represent pictorial/video displays, role plays, recipe making, group sessions, competitions and nutritional assessments.

As shown in Fig 3, intervention activities will be planned as per the villages over a stipulated time period of one year.

#### 1. Provision of training and capacity building of frontline workers and mothers on IYCF practices (SBCC approach).

Training sessions for mothers will be conducted in groups at Anganwadi Centers. These sessions will focus on educating mothers about appropriate weaning foods, preparation of nutritious homemade recipes, feeding frequency, and age-appropriate portion sizes. Additionally, quarterly refresher training will be conducted for both AWWs and mothers to reinforce IYCF knowledge and practices throughout the intervention phase.

SBCC approach will predominantly constitute role plays, community activities, competitions, group sessions and crating role models in community through involvement of gatekeepers.

#### 2. Preparation of food charts, pictorial booklets- AWW, MOTHERS.

A nutritionist from the project team will provide weekly food preparation charts specifically designed for mothers of children aged 6–12 months. These charts will serve as practical guides for mothers.

In addition to the weekly charts, a range of IEC materials will be disseminated for AWWs and mothers to enhance understanding and encourage the adoption of recommended feeding practices. These materials will include illustrated cooking booklets with simple, nutritious recipes, food brochure that can be easily understood regardless of literacy levels. Live cooking demonstrations using locally available ingredients will be organized for mothers and short educational videos will be prepared for demonstrating food preparation techniques, outlining balanced meal plans with emphasis on avoiding processed foods.

To further support learning, live cooking demonstrations will be organized regularly at Anganwadi centres. These sessions will provide hands-on experience to mothers, showing them how to prepare nutritious meals using affordable, seasonal and locally sourced ingredients. Such demonstrations will also be interactive, allowing mothers to ask questions and clarify doubts directly with the nutritionist.

Moreover, mothers will be educated on the importance of avoiding processed and packaged foods that are high in salt, sugar and unhealthy fats. They will be encouraged to prepare fresh, home-cooked meals for their children and guided on recognizing unhealthy food options.

An essential component of this initiative will be the proper maintenance of diet cards. Each mother will be provided with a diet card to record daily feeding details, including the types of food given, quantity and frequency. (S6 File-Diet Card). The nutritionist and field staff will regularly review these cards to monitor progress, provide personalized feedback and ensure adherence to recommended feeding practices.

#### 3. Provision of servings of complementary feeds at anganwadi centres.

Anganwadi Workers (AWWs) will be responsible for preparing age-appropriate solid and semi-solid weaning foods at the Anganwadi Centers, ensuring the provision of hot cooked meals for children aged 6–12 months. Mothers will be encouraged to take two to three servings from the center for their children, while the remaining feeds for the day will be prepared at home. To support this, AWWs will receive provisions of ration supplies for food preparation by the research team.

Each mother will record daily feeding practices in the diet cards provided which will be monitored weekly by research team to track adherence and provide feedback. Mothers demonstrating good compliance and improvement in child feeding may be recognized through prizes and appreciation certificates for AWWs to encourage continued participation.

To facilitate communication and ongoing support, WhatsApp groups will be formed with AWWs and project staff. These groups will be used to share reminders, tips, IEC materials, share photos of recipes and respond to queries, fostering a supportive environment for improving IYCF practices.

#### 4. Monthly examinations of participating infants.

To ensure child health and monitor impact, clinical examination will be carried out by a Medical Officer. These check-ups will include assessments for gastrointestinal and respiratory infections, as well as evaluations of growth patterns through weight and height measurements to determine nutritional status. Children will be screened for MAM, SAM, stunting and underweight conditions using WHO growth standards. Identified cases of SAM and MAM will be treated and referred appropriately.

#### 5. Process Indicators (weekly monitoring).

As shown in Table 2, for ongoing monitoring, weekly process indicators will be recorded, including the number of children receiving at least two complementary feeds at the Anganwadi, number of mothers providing feeds at home (especially on holidays) and those availing feeds from AWCs. Data will also be collected on infants achieving MDD, MMF and MAD, as well as the preparation and use of amylase-rich flour at AWCs. The use of food charts and recipe demonstrations by AWWs, the number of infants consuming iron-rich foods and those receiving iron, calcium and vitamin D supplements will also be tracked. Challenges and gaps identified during the process, along with corrective actions taken, will be documented thoroughly.

**Table 2:** Input, Process Indicators, Outcomes and Impact.

Input	Process	Process Indicators	Outcome Indicators	Impact
• Resources • Training • IEC Material • Replacing strategy of THR to infants in age group of 6–12 months with hot cooked meal	• Training to AWW/ASHA/Mother • Demonstration cooking once in a week for mother • Distribution of charts, booklets and videos • Iron, calcium and vitamin D supplementation • Appropriate and timely weaning at Anganwadi’s	• No. of AWW/ANM trained • No. of mothers trained • no. of demo sessions conducted by AWW • No. of Mothers providing complementary feeds at home on holidays • No. of Mothers availing complementary feeds at Anganwadi’s • No. of Anganwadi’s preparing Amylase rich flour at Anganwadi’s • No. of AWW using food charts, demonstration of preparation of recipes from locally available foods at least monthly for training mothers. • Number of AWW preparing hot cooked food instead of THR for complementary feeding in (6–12 months children) • Number of eligible children in age group 0–12 months who are monitored for growth once in a month.	• Increased in awareness among health care providers and mothers • % of infants who received continued breastfeeding at 1 year • % of infants received solid, semi-solid /soft foods • % of infants achieved MDD, MMF, MAD • Consumption of iron rich foods • % of infants with Improvement in growth parameters, incidence of infections • % of infants received iron, calcium and vitamin D supplements	• % of infants SAM and MAM, stunting, undernutrition • availability of IEC material • sustainability of intervention in program referral system

Abbreviations: THR- Take Home Ration, AWW- Anganwadi Worker, ASHA- Accredited Social Health Activist, ANM- Auxiliary Nurse Midwife, MDD- Minimum Dietary Diversity, MMF- Minimum Meal Frequency, MAD- Minimum Acceptable Diet, SAM- Severe Acute Malnutrition, MAM- Moderate Acute Malnutrition

All this information will be maintained in a dedicated register managed by the research team and health cards for each child will be prepared and updated regularly to support continuous monitoring and evaluation.

### Post-intervention phase (quantitative and qualitative)

A post-intervention survey will be conducted for six months after the intervention phase to evaluate the impact of the intervention on IYCF practices. A total sample of 460 infants aged 6–12 months as per eligibility will be selected from the Ganjad PHC. These may not be same as included for the pre-intervention survey as impact of the interventions in the study area will be assessed. A structured questionnaire as mentioned will be used to collect detailed information on feeding practices, guided by the eight core IYCF indicators. The post-intervention will also include qualitative data collection as done during pre-intervention phase among ASHAs, AWW Supervisor and AWWs by convenient sampling to assess their knowledge and role in promoting appropriate feeding behaviors. The data collection along with the recruitment of participants is expected to be completed by January 2026.

The outcome indicators as shown in Table 2 will be assessed in the post intervention phase. The outcome indicators will specifically measure the percentage of infants who are exclusively breastfed, those who continue breastfeeding up to two years and those who begin receiving complementary foods at six months. It will also assess the proportion of infants who meet the recommended standards for dietary diversity, meal frequency and overall dietary adequacy. Additionally, the study will evaluate the percentage of infants consuming iron-rich foods and receiving essential micronutrient supplements like iron, calcium and vitamin D. An important component of the outcome will also be the percentage of ASHAs and AWWs who demonstrate improved knowledge and awareness of optimal IYCF practices. These outcome measures will help determine the overall effectiveness of the intervention. The projected results are expected to be achieved by February 2026.

### Expected outcome

The expected outcome of the intervention is to achieve a measurable improvement in IYCF practices among the target population in enhancing child nutrition and caregiver practices in the community. The community-based interventions planned in this study will enhance skills of health care workers along with empowering mothers with knowledge and skills thereby leading to improvement in complementary feeding practices which may result in eventual reduction in prevalence of SAM, MAM, undernutrition in the area. This model can be scaled up in other blocks of Palghar district and also in other tribal areas of India.

### Data collection tool and statistical analysis plan

Data will be collected using a pre-tested structured questionnaire and recorded systematically. The collected data will be entered into Microsoft Excel and subsequently imported into Statistical Package for the Social Sciences (SPSS) software for analysis. Data cleaning and validation will be performed prior to analysis to ensure accuracy and completeness.

Descriptive statistics such as frequencies, percentages, means, and standard deviations will be used to summarize socio-demographic characteristics and key study variables. Inferential statistical tests (such as Chi-square test for categorical variables and t-test for continuous variables) will be applied to compare pre- and post-intervention outcomes. Regression analysis will be used to control for confounding factors. Statistical significance will be considered at p < 0.05.

Qualitative data (Nvivo version-13) will be used for analysis of qualitative data. Data obtained from in-depth interviews will be transcribed verbatim and analyzed separately using 3thematic analysis. Codes and themes will be developed to identify key patterns, perceptions, and challenges related to IYCF practices and intervention implementation.

### Dissemination plan

The findings of the study will be disseminated through a multi-pronged strategy to ensure that all key stakeholders, including policy-makers, health professionals and community workers, are informed and engaged.

## Discussion

The study proposes a comprehensive targeted community-based approach to improve IYCF practices among tribal populations in Palghar District, Maharashtra. The available evidence has highlighted the gaps between recommended and current IYCF practices, necessitating need of strengthening and upgrading skills of the existing healthcare staff by making them more resourceful to improve the nutritional status of the children in their respective communities [20,21]. However, the present study builds upon previous experience of authors in same tribal block demonstrating suboptimal IYCF practices and impact of multicomponent intervention on nutrition of children under-five [15]. However, going a step ahead, the current study lays out specific strategies to enhance IYCF practices in tribal region of Palghar district.

While national initiatives like ICDS and POSHAN Abhiyan of India seek to promote child nutrition, implementation in tribal areas is frequently hampered by logistical difficulties, cultural traditions, low literacy and a lack of community engagement. Despite significant initiatives by Government of India to improve the nutritional status of under-five children, a large proportion of them are still malnourished and there is a need to improve their nutritional status through specific targeted interventions. Our study plans integrating SBCC strategy with provision of hot cooked meal at Anganwadi’s through involvement of frontline workers to address multiple facets of potential implementation challenges.

Under the ICDS programme, children aged 3–6 years receive nutrition in the form of hot cooked meals while children under three years of age receive THR (‘Take home ration’). However, studies have shown that cooked food is a more effective strategy in providing supplementary nutrition than THR packets since it is more acceptable, consumed, and has an adequate amount of calories and protein [22].

Notably, our proposed intervention of providing hot cooked meal at Anganwadi’s is in accordance to recommendations highlighted from earlier experiences from some of the states and tribal regions of Maharashtra where better acceptance and utilization of fresh hot cooked food to children below the age of 3 years will be demonstrated [23,24]. Moreover, the emphasis on locally available food, visual educational aids, live recipe demonstrations and tracking feeding practices will develop a culturally sensitive and feasible framework from perspective of sustainability.

Furthermore, capacity building of AWWs and ASHAs, developing role models in community will enhance community engagement. Moreover, tribal populations in India are affected by a trio of primary concerns including cultural impediments, socioeconomic difficulties and inaccessibility to the health system due to geographical disparities. This comprehensive strategy highlights frontline workers as village ambassadors for improved IYCF practices which may eventually translate into better anthropometric outcomes of tribal children.

The study puts forward a scalable and replicable strategy by focussing on critical period of first two years (730 days) of life contributing to “Journey first 1000 days” of life aligning with objectives of WHO-UNICEF global nutrition target to improve nutritional outcomes of infants [25].

Additionally, the intervention may prove as feasible and long-term sustainable approach if adapted within existing ICDS program through utilization of allotted rations, locally available foods and existing resources. However, future assessment of cost effectiveness of this proposed intervention with the existing intervention may provide better insights for integration in the program through engagement of local stakeholders.

This study will address the need for context-specific interventions to enhance IYCF practices in tribal regions of India. The proposed model encompassing behaviour change communication, hot cooked meal provision at Anganwadi centers and empowering community may offer a sustainable and expandable solution to improve nutrition in the first two years of life.

Importantly, by leveraging potential of frontline health care workers to enhance IYCF practices in community, the study can pave a path for transforming child nutrition in tribal blocks. Importantly, the findings may provide crucial evidence for policy improvements and targeted interventions within ICDS and POSHAN 2.0 frameworks.

## Supporting information

S1 File Supporting File 1- TREND checklist. (DOCX)

S2 File Supporting File 2- COREQ checklist. (DOCX)

S3 File Supporting File 3- In Depth Interview Guide. (DOCX)

S4 File Supporting File 4- Data Collection tool -Mothers and Children. (DOCX)

S5 File Supporting File 5- Complementary feeding guidelines. (DOCX)

S6 File Supporting File 6- Diet card. (DOCX)

## Abbreviations

ANM: auxiliary nurse midwife
IYCF: infant and young child feeding practices
MAD: minimum acceptable diet
MAM: moderate acute malnutrition
MDD: minimum dietary diversity
MMF: minimum meal frequency
PHC: primary health center
SAM: severe acute malnutrition
THR: take home ration
WHO: world health organization.
