# Supplementary material for: Protocol for a quasi experimental mixed method study on impact of intervention for improving Infant and Young Child Feeding (IYCF) practices in tribal block of Palghar District, Maharashtra, India through involvement of frontline workers
Source: PLoS One. 2026 Jul 15;21(7):e0353241. doi: 10.1371/journal.pone.0353241 (PMC13372156; doi:10.1371/journal.pone.0353241)
Supplement: S2 File — (DOCX) [file pone.0353241.s002.docx]

**Consolidated criteria for reporting Qualitative research checklist**

| **Topic** | **Item No.** | **Guide Questions/Description** | **Reported on**  **Page No.** |
| --- | --- | --- | --- |
| **Domain 1: Research team and reﬂexivity** | | | |
| *Personal characteristics* | | | |
| Interviewer/facilitator | 1 | Which author/s will conduct the interview or focus group? | 481-485 |
| Credentials | 2 | What are the researcher’s credentials? E.g. PhD, MD | 7-23 |
| Occupation | 3 | What will be their occupation at the time of the study? | 7-23 |
| Gender | 4 | Will the researcher male or female? | 4-6 |
| Experience and training | 5 | What experience or training did the researcher have? | 7-23 |
| *Relationship with participants* | | | |
| Relationship established | 6 | Will a relationship be established prior to study commencement? | 214-244 |
| Participant knowledge of the interviewer | 7 | What will the participants know about the researcher? e.g. personal goals, reasons for doing the research | 184-196 |
| Interviewer characteristics | 8 | What characteristics will be reported about the inter viewer/facilitator?  e.g. Bias, assumptions, reasons and interests in the research topic | 184-196 |
| **Domain 2: Study design** | | | |
| *Theoretical framework* | | | |
| Methodological orientation and Theory | 9 | What methodological orientation will be stated to underpin the study? e.g. grounded theory, discourse analysis, ethnography, phenomenology, content analysis | 117-122 |
| *Participant selection* | | | |
| Sampling | 10 | How will participants be selected? e.g. purposive, convenience,  consecutive, snowball | 124-178 |
| Method of approach | 11 | How will participants be approached? e.g. face-to-face, telephone, mail, email | 124-178, 184-196 |
| Sample size | 12 | How many participants will be in the study? | 157-168 |
| Non-participation | 13 | How many people will be refuse to participate or drop out? Reasons? | 184-196 |
| *Setting* | | | |
| Setting of data collection | 14 | Where will the data be collected? e.g. home, clinic, workplace | 124-155 |
| Presence of non-  participants | 15 | Will anyone else be present besides the participants and researchers? | 184-196 |
| Description of sample | 16 | What are the important characteristics of the sample? e.g. demographic data, date | 223-244 |
| *Data collection* | | | |
| Interview guide | 17 | Will questions, prompts, guides be provided by the authors? Will it be pilot tested? | 223-244 |
| Repeat interviews | 18 | Will repeat interviews be carried out? If yes, how many? | NA |
| Audio/visual recording | 19 | Will the research use audio or visual recording to collect the data? | 184-196 |
| Field notes | 20 | Will field notes be made during and/or after the interview or focus group? | 223-244 |
| Duration | 21 | What will be the duration of the interviews or focus group? | 198-201 |
| Data saturation | 22 | Will data saturation be discussed? | NA |
| Transcripts returned | 23 | Will transcripts be returned to participants for comment and/or | 184-196 |
