# Supplementary material for: Protocol for a quasi experimental mixed method study on impact of intervention for improving Infant and Young Child Feeding (IYCF) practices in tribal block of Palghar District, Maharashtra, India through involvement of frontline workers
Source: PLoS One. 2026 Jul 15;21(7):e0353241. doi: 10.1371/journal.pone.0353241 (PMC13372156; doi:10.1371/journal.pone.0353241)
Supplement: S3 File — (DOCX) [file pone.0353241.s003.docx]

**Supporting File- 3-In Depth Interview Guide**

**Supporting File- 3.1. Baseline Survey (ASHA/AWW) (PHC/SC)**

Name: Gender: Age:

Designation: Place of work:

1. Number of children enrolled in Anganwadi
2. Number of children in age group of 6 to 12 months Anganwadi
3. In your opinion, when breast feeding should be initiated
4. How long Breast feeding should be given
5. Do you thing colostrum should be given to baby
6. Do you advise supplementary feeds (water/ animal milk/honey/Buti) along with breast feed
7. When complementary feeding should be started
8. How long breast feeding should be continued
9. Which food you suggest to include as complementary feeds
10. What should be frequency of complementary feeds
11. Have you heard about different food groups
12. What are min food groups to be included in complementary feeds
13. Can animal milk be started as complementary feed
14. Have you heard of amylase rich flour
15. Do you prepare such flour at your Anganwadies
16. Do you use sprouted grains in Anganwadi foods
17. Do you receive THR regularly
18. Do you feel utilization of THR is appropriately done by mothers
19. Where do you refer children having SAM
20. Where do you refer children having MAM
21. Do you feel weaning practices are proper in your area
22. Do you receive regular incentives for your work
23. How often RBSK team visits your center
24. What are iron rich foods
25. What are Calcium rich foods
26. What are your Challenges

**Supporting files 3.2: In Depth Interview Guide-AWW**

Name: Gender: Age:

Designation: Place of work:

1. Components of IYCF Practices
2. Importance of exclusive breast feeding
3. Initiation of complementary feeding
4. Continuation of breast feeding
5. Distribution of THR
6. Do you feel utilization of THR is appropriately done by mothers
7. Current strategy of management of SAM and MAM children in Anganwadi’s
8. Frequency of RBSK team visits
9. Distribution of Iron, Calcium and Vitamin D supplements
10. What are your Challenges and barriers in implementation of ICDS program at Anganwadi’s

**Supporting File 3.3: In Depth Interview for ASHA workers**

Name: - Age- Village Name: -

Education: - Mobile no: -

1. How many pada do you have as an ASHA worker, and what is their population
2. In how many Anganwadi do you work and what is the nature of your work in Anganwadi?
3. What is your role in the community? What work do you do for community?
4. What difficulties do you face while doing this task?
5. What work do you do related to the Public Health?
6. Explain the work on pregnant women lactating women and malnourished children?
7. Why is it necessary to breastfeed the baby?
8. What foods are included in complementary diet?
9. Do you think mothers use THR appropriate?
10. Do you think breastfeeding practices are appropriate in your community?
11. Which IEC Materials would be useful if complementary feeding practice were to be improved?

**Supporting File 3.4: In Depth Interview (AWW)**

Name: Gender: Age:

Designation: Place of work:

1. What foods are suitable for infants and young children?

Top of Form

1. What is the importance of breastfeeding? Why is breastfeeding necessary?
2. From which month should complementary feeding be introduced to children, and what can be given as complementary feeding?
3. Until what age should breastfeeding be provided to children, and why is it important to continue breastfeeding?
4. How is the distribution of THR done?
5. Do you think mothers use THR appropriately?
6. What is the current plan for the management of SAM (Severe Acute Malnutrition) and MAM (Moderate Acute Malnutrition) children in Anganwadi’s? What challenges do you face under these plans?
7. How often RBSK team visits your center and how is their planning done?
8. What is the sources of Iron, Calcium, and Vitamin D?
9. What programs are conducted in Anganwadi’s, and what challenges do you face in them?
10. What kind of diet is provided to children aged 3 months to 6 years in anganwadi?
11. How do you receive honorarium/salary in Anganwadi’s?
12. What interventions are available for lactating and pregnant women in Anganwadi’s?
13. What is Amylase-rich flour, and how is it prepared?
14. Can cow's milk be given to children under six months, and can it be given as complementary feeding?

**Supplementary File 1.5.: In Depth Interview of CPDO**

Name: Gender: Age:

Designation: Place of work:

1. Components of IYCF Practices
2. Importance of Exclusive breast feeding
3. Initiation of complementary feeding
4. Continuation of Breast feeding
5. Distribution of THR
6. Do you feel utilization of THR is appropriately done by mothers?
7. Current strategy of management of SAM & MAM children in Anganwadi
8. Frequency of RBSK team visits
9. Distribution of Iron, Calcium & Vitamin D supplements
10. What are your challenges & barriers in implementation of ICDS program at Anganwadi?
