# Supplementary material for: Protocol for a quasi experimental mixed method study on impact of intervention for improving Infant and Young Child Feeding (IYCF) practices in tribal block of Palghar District, Maharashtra, India through involvement of frontline workers
Source: PLoS One. 2026 Jul 15;21(7):e0353241. doi: 10.1371/journal.pone.0353241 (PMC13372156; doi:10.1371/journal.pone.0353241)
Supplement: S4 File — (DOCX) [file pone.0353241.s004.docx]

**Supporting File 4.1: Questionnaires for Mothers**

**Improving Infant and young child Feeding (IYCF) practices in Tribal block of Palghar District, Maharashtra through involvement of frontline workers**

| **1.** | **Village (_______________________)** |  |  | **5.** | **Date of Survey:**  **D D M M Y E A R**   \|  \|  \|  \|  \|  \|  \|  \|  \| \| --- \| --- \| --- \| --- \| --- \| --- \| --- \| --- \| | |
| --- | --- | --- | --- | --- | --- | --- | --- | --- | --- | --- | --- | --- | --- | --- |
| **2.** | **District (_______________________)** |  |  | **6.** | **Name of the mother: ___________________** | |
| **3.** | **AWC Code (______________________)** |  |  | **7.** | **Age at marriage (Yrs): __________________** | |
| **4.** | **Number of Live Births (____________)** |  |  | **8.** | **Participant No:**   \|  \|  \|  \| \| --- \| --- \| --- \| | |
|  |  |  |  |  |  |  |

| **Particulars of Child** | | | |
| --- | --- | --- | --- |
| Name of the Index Child: ____________________ | | | |
| Date of birth :__ __ / __ __ / __ __ | | | |
| 1. | | Age (in completed months) :__ __ | |
| 2. | | Gender  (1. Male 2. Female) | : __ |
| 3. | | Birth order | : __ |
| 4. | | If birth order is >1, interval between the last two live births (months) : __ __ | |
| **Current Feeding Practices** | | | |
| 5. | | Type of feeding being given currently?  (1. Yes 2. No 9. NA)  1. Only breast milk  2. Breast milk + water  3. Breast milk + complementary feeds  4. Bottle feeding 5. Normal food | : __ |
| 6. | | What should be the immediate feed to be given to a child after birth  (1. Yes 2. No 9. NA)  1. Breast feed  2. Water  3. Honey  4. Cow’s milk  5. Others | : __ |
| 7. | | How long only breast feeding should be given to a child? |  |
| 8. | | Up to what age (months) the child was given only breast-milk  (Without water)?  (Enter 99 if still on only breastfeeding) |  |
| 9. | | At what age complementary feeding should be initiated? |  |
| 10. | | What foods should be included in complementary feed?  (1. Yes 2. No 9. NA)  1. Cow / buffalo milk  2. Formula milk 3. ICDS Supplement (____________)  4. Commercial baby foods  5. Processed Foods (Biscuits etc.)  6. Homemade semi-solids  7. Homemade solids  8. Cereals & millets  9. Pulses 10. GLV 11. Other Vegetables  12. Roots & tubers 13. Fruits 14. Milk & milk products  15. Eggs 16. Meat/fish/chicken  17. Fats & Oils 18. Sugar & jaggery  19. Other |  |
| 11. | | How many times complementary feed should be given in a day? | : __ |
| 12. | | How long exclusive breast feeding should be given? | : __ |
|  |  | And how long it should be continued? | : __ |
| 13. | | When did you start giving complementary food to your child? (Months) (Enter ‘99’ if not started) | : __ |
| 14. | | Up to what age (months) the child was given breast-milk?  (Enter 99 if still on breastfeeding) |  |
| 15. | | Type of complementary foods currently given?  (1. Yes 2. No 9. NA)  1. Cow / buffalo milk  2. Formula milk 3. ICDS Supplement (____________)  4. Commercial baby foods  5. Processed Foods (Biscuits etc.)  6. Homemade semi-solids  7. Homemade solids  8. Others |  |
| 16. | | No. of complementary feeds/day **given by you?** |  |
| 17. | | What food was given to the child during the previous day apart from breast milk? (____________________________) |  |
| 18. | | Generally, how do you feed complementary food to the child  (1. Yes 2. No 9. NA)  1.With spoon  2. With hand  3. Self with spoon  4. Self by hand  5. Bottle feeding |  |
| **Participation in ICDS Programme** | | | |
| 19. | For ho many days did you take THR for child in last month?  1.≥ 20 days/month  2. <20 days/month  3. Not taking | |  |
| 20 | How many and which THR packet did you received?  (1. Yes 2. No 9. NA)  1.Multi mix cereals and protein premix (Green packet)  2.Moongdal khichadi premix (yellow packet)  3.Turdal khichdi premix (Pink packet) | |  |
| 21. | . If not taking, give reasons  1.AWC not accessible  2. No need 3. Not Offered  4. SNP not good 5. Causing ill health  6. Social Hierarchy 7. Others (Specify) 9. NA | |  |
| 22. | If THR is received, is the supplement shared among family members?  1. Yes 2. No 9. NA | |  |
| **IFA Tablets/Syrup distribution to Children**  **(Show the Sample Tablets/Syrup bottle)** | | | |
| 23. | Did your child receive the following syrup/tablet in last one year?  (1. Yes 2. No 9. NA)  1. IFA  2. Vitamin-A  3. Vitamin-D  4.Any Other | |  |
| **Care of the Child** | | | |
| 24. | Generally, who looks after the child  when you go out for work?  (1. Yes 2. No 9. NA)  1. Mother-in-law  2. Father-in- law  3. Elder siblings  4. Left at AWC/ Crèche  5. Carry the child to work spot  6. Other  9. NA | |  |

25. Do you know about Calcium Rich Foods 1. Aware 2. Not aware

| Food | 1.Yes 2.No | Daily | Once in a week | 2-3 times in a week | Occasionally |
| --- | --- | --- | --- | --- | --- |
|  |  |  |  |  |  |
| Milk |  |  |  |  |  |
| Cheese/Paneer |  |  |  |  |  |
| Green Vegetables |  |  |  |  |  |
| Banana |  |  |  |  |  |
| Ragi |  |  |  |  |  |
| Health drink |  |  |  |  |  |
| Supplementation |  |  |  |  |  |

26. Do you know about Vitamin D rich Foods 1. Aware 2. Not aware

| Food | 1.Yes 2.No | Daily | Once in a week | 2-3 times in a week | Occasionally |
| --- | --- | --- | --- | --- | --- |
| Milk |  |  |  |  |  |
| Egg yolk |  |  |  |  |  |
| Cheese |  |  |  |  |  |
| Oily Fishes |  |  |  |  |  |
| Health drink |  |  |  |  |  |
| Supplementation |  |  |  |  |  |

27. Do you know about Iron rich Foods 1. Aware 2. Not aware

| Food | 1.Yes 2.No | Daily | Once in a week | 2-3 times in a week | Occasionally |
| --- | --- | --- | --- | --- | --- |
|  |  |  |  |  |  |
| Whole grain |  |  |  |  |  |
| Fruits |  |  |  |  |  |
| Leafy Green-vegetables |  |  |  |  |  |
| Red meat |  |  |  |  |  |
| Health drink |  |  |  |  |  |
| Supplementation |  |  |  |  |  |

28. Weight (Kg): _________________ 29. Height (cm): ______________________

**Signature of Project Technical Support III with date Signature of Field worker with date**

**Name: _______________________________________ Name:_________________________**

**Supporting File 4.2: Case Record Form**

**Improving Infant and young child Feeding (IYCF) practices in Tribal block of Palghar District, Maharashtra through involvement of frontline workers**

| **A** | **Participant No.** | | | |  | |  | |  | | | | | | |  |
| --- | --- | --- | --- | --- | --- | --- | --- | --- | --- | --- | --- | --- | --- | --- | --- | --- |
|  |  | | | |  | |  | | |  | | |  | | | |
| A1 | 1. Pre intervention 2. Post Intervention | | | |  | |  | | |  | | |  | | | |
|  | D D M M Y E A | | | | | | | | | | | | | | R | |
| A2 | Date |  |  |  | |  |  |  | | | |  | | |  | |
|  |  |  |  |  | |  |  |  | | | |  | | |  | |
| A3 | Parents phone number |  |  |  | |  |  |  | | | |  | | |  | |
|  |  |  |  |  | |  |  |  | | | |  | | |  | |
| A4 | Age in years and months | | | | | | | | | | |  | | |  | |
|  |  | | | | | | | | | | |  | | |  | |
| A5 | Birth Order | | | | | | | | | | |  | | |  | |
|  |  | | | | | | | | | | |  | | |  | |
| A6 | Gender: 1. Male 2. Female | | | | | | | | | | |  | | |  | |
|  |  | | | | | | | | | | |  | | |  | |
| A7a | Name of Mother ______________________________________________________ | | | | | | | | | |  | | | | | |
|  |  | | | | | | | | | | | | |  | | |
| A7b | Name of Father ______________________________________________________ | | | | | | | | | | | | |  | | |

| **B** | **Birth History** | |  |
| --- | --- | --- | --- |
| B1 | Antenatal problems 1. Yes 2. No |  | |
|  |  |  | |
| B2 | If Yes, 1. PIH 2. GDM 3. Leaking PV 4. TB 5. Blood disorders 6. Other 99. NA |  | |
|  |  |  | |
| B3 | If Other, specify |  | |
|  |  | | |
| B4 | Gestational age: 1. GA <37 Weeks 2. >37 weeks; |  | |
|  |  |  | |
| B5 | If < 37 weeks then how many weeks |  | |
|  |  |  | |
| B6 | Mode of delivery: 1. Normal 2. LSCS 3. Instrumentation |  | |
|  |  |  | |
| B7 | If LSCS, Indication: |  | |
|  |  |  | |
| B8  B9 | Birth weight: ___  IUGR 1. Yes 2. No |  | |
|  |  |  | |
|  |  |  | |
| B10 | Cried Immediately after birth (H/O Birth asphyxia):1. Yes 2. No |  | |
|  |  |  | |
| B11 | Congenital anomalies 1.Yes 2.No |  | |
|  |  |  | |
| B12 | If Yes, Specify |  | |
|  |  |  | |
| B13 | NICU Stay 1. Yes 2.No |  | |
|  |  |  | |
|  | **If yes, details_________** |  | |
| B14 | Family History of Haemoglobinopathies (1. Yes 2. No) |  | |
|  |  |  | |
|  | Past History of hospital admission (1. Yes 2. No) |  |  |
|  |  |  | |
|  | If yes, details_________ |  |  |
|  |  |  |  |
| B15 | Number of acute respiratory tract infection since birth |  | |
|  |  |  |  |
|  | Number of acute Gastro intestinal tract infection since birth |  | |

|  | | | | | | | | | | | | | | |  |  |  |  |  |
| --- | --- | --- | --- | --- | --- | --- | --- | --- | --- | --- | --- | --- | --- | --- | --- | --- | --- | --- | --- |
|  |  |  |  |  |  |  |  |  |  |  |  |  |  |  |  |  |  |  |  |
| C1 | Weight in Kg | | | | | | | | | | |  | | |  |  |  |  |  |
|  |  | | | | | | | | | | | | | |  |  |  |  |  |
| C2 | Length/Height in cms | | | | | | | | | |  |  | | |  |  |  |  |  |
|  |  | | | | | | | | | | | | | |  |  |  |  |  |
| C3 | Head circumference in cms | | | | | | | | | | |  | | |  |  |  |  |  |
|  |  | | | | | | | | | | | | | |  |  |  |  |  |
| C4 | Mid arm circumference in cms | | | | | | | | | | |  | | |  |  |  |  |  |
|  |  | | | | | | | | | | | | | |  |  |  |  |  |
| C5 | Growth 1. < 3 SD (SAM) 2. -2SD-3SD (MAM) 3.>2SD (Normal) | | | | | | | | | | | | | |  |  |  |  |  |
|  |  | | | | | | | | | | | | | |  |  |  |  |  |
| C6 | Immunization History complete 1. Yes 2. No 99.NA | | | | | | | | | | | | | |  |  |  |  |  |
|  | If no____________ | | | | | | | | | | | | | |  |  |  |  |  |
| C7 | Milestones: 1. Normal 2. Abnormal  If abnormal_____________________ | | | | | | | | | | | | | |  |  |  |  |  |
|  |  | | | | | | | | | | | | | |  |  |  |  |  |
|  |  | | | | | | | | |  | | | | |  |  |  |  |  |
| **D** | **Clinical Examination** | | | | | | | | | | | | | |  |  |  |  |  |
|  |  | | | | | | | | | | | | | |  |  |  |  |  |
| D1 | Pallor: 1. Nil 2. Mild 3. Moderate 4. Severe | | | | | | | | | | | | | |  |  |  |  |  |
|  |  | | | | | | | | | | | | | |  |  |  |  |  |
| D2 | Icterus 1. Yes 2. No | | | | | | | | | | | | | |  |  |  |  |  |
|  |  | | | | | | | | | | | | | |  |  |  |  |  |
|  |  | | | | | | | | | | | | | |  |  |  |  |  |
| D3 | Heart rate |  | Heart rate | | | | | | | | | | | |  |  |  |  |  |
|  |  | | | | | | | | | | | | | |  |  |  |  |  |
| D5 | Frontal Bossing 1. Yes 2. No | | | |  | | | Frontal Bossing 1. Yes 2. No | | | | | | | | | | | |
|  |  | | | |  | | | | | | | | | |  |  |  |  |  |
|  |  | | | | | | | | | | | | | |  |  |  |  |  |
| **E** | **Systemic Examination** | | | | | | | |  | | | | | | | |  |  |  |
| E1 | **Resp System** | | |  | | |  | | | | | | |  | | |  |  |  |
|  |  |  |  |  | | |  |  |  |  |  |  |  |  | | |  |  |  |
|  |  | | | | | | | | | | | | | | | | |  | |
|  |  | | | | | | | | | | | |  | | |  | | |  |
|  | **Per Abdomen** 1. Yes 2. No | |  | | |  | | | | | | |  | | |  |  |  |  |

| E2 | CNS 1. Normal 2. Abnormal |  |  | |  |
| --- | --- | --- | --- | --- | --- |
|  |  | | |  | |
| E3 | CVS 1. Normal 2. Abnormal |  |  | |  |

**Signature of Scientist-I with date**

**Name: _______________________**

***Clinical Notes:***
