# Supplementary material for: Protocol for a quasi experimental mixed method study on impact of intervention for improving Infant and Young Child Feeding (IYCF) practices in tribal block of Palghar District, Maharashtra, India through involvement of frontline workers
Source: PLoS One. 2026 Jul 15;21(7):e0353241. doi: 10.1371/journal.pone.0353241 (PMC13372156; doi:10.1371/journal.pone.0353241)
Supplement: S5 File — (DOCX) [file pone.0353241.s005.docx]

**Supporting File 5.1: Complementary feeding guidelines**

| **Age** | **Texture** | **Frequency** | **Average amount each meal** |
| --- | --- | --- | --- |
| 6-8 months | Start with thick porridge, well mashed foods | 2-3 meals per day plus frequent BF | Start with 2-23 table spoonful |
| 9-11 months | Finely chopped or mashed foods, and foods that baby can pick up | 3-4 meals plus BF. Depending on appetite offer 1-2 snacks | ½ of a 250ml cup/bowl |
| 12-23 months | Family foods, chopped or mashed if necessary | 3-4 meals plus BF. Depending on appetite offer 1-2 snacks | ¾ to one 250ml cup/bowl |

**Abbreviations: BF- Breastfeeding**

**Supporting File 5.2 Feeding guide**

| **Age** | **Feeding Recommendation** |
| --- | --- |
| 6-12 months | - Continue breastfeeds on demand. Sequential transition of diet and nutrition takes place during this phase: - Amount: offer ¼- ½ - ¾ -1 katori/ serving - Frequency: 3-4 times /day if breast fed; 5-6 times/day with no breast feed - Offer variety of food with added oil/ghee. - Transition from soft mashed blend food to grainy thick food to family food – suji/ ragi(*nagli*)/ thick pasty washed dal then to blended mix veg soup to kichadi with soft cooked mashed vegetable to mashed roti in dal or milk - Roti with dal or milk/ *dalia* with milk/ locally available fruit piece/ boiled sweet potato or carrot sticks to cooked vegetables from family pot - Mother and child should wash their hands with soap and water every time before feeding. |
| 12-24 months | - Breastfeed on demand - Offer food from the family pot- 5times/day - At-least 1 ½ katori serving at a time - Snack: paushtik ladoo, chapati ladoo - Mother and child should wash their hands with soap and water every time before feeding. |

**Supporting File 5.3: Number of Portions for Infant and Young children**

| **Food groups** | **g/portion** | **Infants 6-12months** | **1-3 years** |
| --- | --- | --- | --- |
| Cereal and Millets | 30 | 0.50 | 2 |
| Pulse | 30 | 0.25 | 1 |
| Milk (ml) | 100 | 4 | 5 |
| Roots and Tuber | 100 | 0.50 | 0.5 |
| Green Leafy Vegetable | 100 | 0.25 | 0.5 |
| Other Vegetable | 100 | 0.25 | 0.5 |
| Fruits | 100 | 1 | 1 |
| Sugar | 5 | 2 | 3 |
| Fats and Oil (Visible) | 5 | 4 | 5 |

**Source:** Dietary Guideline for Indian-A Manual. NIN, ICMR.2011
