# Supplementary figures and images for: Protocol for a quasi experimental mixed method study on impact of intervention for improving Infant and Young Child Feeding (IYCF) practices in tribal block of Palghar District, Maharashtra, India through involvement of frontline workers
Source: PLoS One. 2026 Jul 15;21(7):e0353241. doi: 10.1371/journal.pone.0353241 (PMC13372156; doi:10.1371/journal.pone.0353241)

**Supporting File 6: Diet Card**


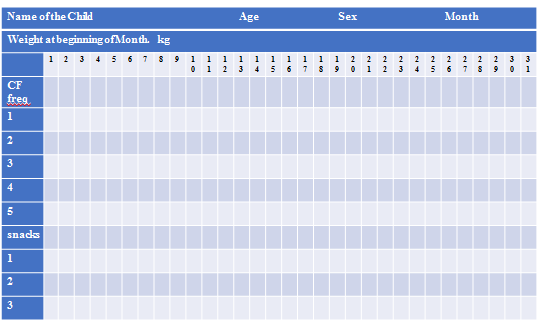


**Abbreviations: CF- Complementary feeding, freq- Frequency**

Supplement: S6 File — (DOCX) [file pone.0353241.s006.docx]
