# Supplementary material for: Protocol for a quasi experimental mixed method study on impact of intervention for improving Infant and Young Child Feeding (IYCF) practices in tribal block of Palghar District, Maharashtra, India through involvement of frontline workers
Source: PLoS One. 2026 Jul 15;21(7):e0353241. doi: 10.1371/journal.pone.0353241 (PMC13372156; doi:10.1371/journal.pone.0353241)
Supplement: Attachment — Submitted filename: PONE-D-25-38092_reviewed_Elias.pdf [file pone.0353241.s008.pdf]

PLOS ONE

Protocol for Study on Improving Infant and Young Child Feeding (IYCF) Practices in Tribal Block of Palghar District, Maharashtra through Involvement of Frontline Workers

--Manuscript Draft--

**Manuscript Number:**	PONE-D-25-38092
**Article Type:**	Study Protocol
**Full Title:**	Protocol for Study on Improving Infant and Young Child Feeding (IYCF) Practices in Tribal Block of Palghar District, Maharashtra through Involvement of Frontline Workers
**Short Title:**	Improving Infant and Young Child Feeding (IYCF) Practices in Tribal Block of Palghar District Maharashtra
**Corresponding Author:**	Ragini Kulkarni, M.D<br>NIRRCH: National Institute for Research in Reproductive and Child Health<br>Mumbai, Maharashtra INDIA
**Keywords:**	Keywords: Undernutrition, IYCF, complementary feeding, weaning, tribal children
**Abstract:**	Introduction: Infant and Young Child Feeding (IYCF) is crucial for child growth, especially in the first two years. Despite efforts, malnutrition remains high 35.5% of under-five children in India are stunted (2023). In Maharashtra, tribal areas like Palghar are most affected due to poverty, poor healthcare, and low awareness. Anganwadi Workers (AWWs) are key to IYCF promotion but face significant challenges. The study aims to assess the knowledge, attitude, and practices (KAP) of healthcare workers and mothers on IYCF. Based on the findings, an intervention package will be developed to address gaps and improve IYCF practices. The study will also compare children's nutritional status and key WHO IYCF indicators before and after the intervention, and document facilitating factors and challenges during its implementation.<br>Methods: A quasi-experimental study will be conducted over two years in three phases in the Ganjad PHC area in the tribal region of Palghar District, Maharashtra. Based on prior findings, 460 children will be enrolled each for the pre- and post-intervention surveys. Sampling will be done across 44 Anganwadi centers, with 10–11 eligible children selected per center. Infants aged 6–12 months, their mothers, and randomly selected ASHAs and AWWs will be included for In-depth interviews. Children with chronic illnesses, Severe Acute Malnutrition/Moderate Acute Malnutrition, birth defects, or those whose mothers do not provide written informed consent will be excluded.<br>Discussion: This study proposes a comprehensive, community-based intervention to improve IYCF practices among tribal populations in Palghar District, Maharashtra. It integrates behavior change communication with hot cooked meal provision at Anganwadi centers, leveraging frontline workers for implementation. The approach emphasizes cultural sensitivity, sustainability, and local resource utilization. Findings may offer scalable solutions and inform policy within ICDS.<br>Clinical trial registration: CTRI/2024/06/068427 Registered on 06th June, 2024.
**Order of Authors:**	Suchitra Surve
	Shraddha Kyatam
	Shahina Begum
	Bhavya M K
	Santosh Chaudhari
	Sagar Patil
	Shivshankar Timmanpyati
	Purabi Mahajan
	Ragini Kulkarni
**Additional Information:**	
**Question**	**Response**

Powered by Editorial Manager® and ProduXion Manager® from Aries Systems Corporation

**Financial Disclosure**<br><br>Enter a financial disclosure statement that describes the sources of funding for the work included in this submission. Review the submission guidelines for detailed requirements. View published research articles from *PLOS ONE* for specific examples.<br><br>This statement is required for submission and **will appear in the published article** if the submission is accepted. Please make sure it is accurate.<br><br>**Funded studies**<br>Enter a statement with the following details:<br>• Initials of the authors who received each award<br>• Grant numbers awarded to each author<br>• The full name of each funder<br>• URL of each funder website<br>• Did the sponsors or funders play any role in the study design, data collection and analysis, decision to publish, or preparation of the manuscript?<br><br>Did you receive funding for this work?	Yes; Yes
Please add funding details.<br>as follow-up to "**Financial Disclosure**<br><br>Enter a financial disclosure statement that describes the sources of funding for the work included in this submission. Review the submission guidelines for detailed requirements. View published research articles from *PLOS ONE* for specific examples.<br><br>This statement is required for submission and **will appear in the published article** if the submission is accepted. Please make sure it is accurate.	Initials of the authors: SS<br>Grant number: (CTU/PM ABHIM/17/10/46/2023-ECD-II).<br>Full Name of Funder: Indian Council of Medical Research (ICMR).<br>The funders did not play any role in the study design, data collection and analysis, decision to publish, or preparation of the manuscriptIndian Council of Medical Research (ICMR) (CTU/PM ABHIM/17/10/46/2023-ECD-II).

**Funded studies**<br>Enter a statement with the following details:<br>• Initials of the authors who received each award<br>• Grant numbers awarded to each author<br>• The full name of each funder<br>• URL of each funder website<br>• Did the sponsors or funders play any role in the study design, data collection and analysis, decision to publish, or preparation of the manuscript?<br><br>Did you receive funding for this work?"	
Please select the country of your main research funder (please select carefully as in some cases this is used in fee calculation).<br>as follow-up to "**Financial Disclosure**<br><br>Enter a financial disclosure statement that describes the sources of funding for the work included in this submission. Review the submission guidelines for detailed requirements. View published research articles from *PLOS ONE* for specific examples.<br><br>This statement is required for submission and **will appear in the published article** if the submission is accepted. Please make sure it is accurate.<br><br>**Funded studies**<br>Enter a statement with the following details:<br>• Initials of the authors who received each award<br>• Grant numbers awarded to each author<br>• The full name of each funder<br>• URL of each funder website<br>• Did the sponsors or funders play any role in the study design, data collection and analysis, decision to publish, or preparation of the manuscript?<br><br>Did you receive funding for this work?"	INDIA - IN; INDIA - IN
**Competing Interests**	The authors have declared that no competing interests exist.

Use the instructions below to enter a competing interest statement for this submission. On behalf of all authors, disclose any competing interests that could be perceived to bias this work—acknowledging all financial support and any other relevant financial or non-financial competing interests.<br><br>This statement is required for submission and **will appear in the published article** if the submission is accepted. Please make sure it is accurate and that any funding sources listed in your Funding Information later in the submission form are also declared in your Financial Disclosure statement.<br><br>View published research articles from *PLOS ONE* for specific examples.<br><br>**NO authors have competing interests**<br><br>Enter: *The authors have declared that no competing interests exist.*<br><br>**Authors with competing interests**<br><br>Enter competing interest details beginning with this statement:<br><br>*I have read the journal's policy and the authors of this manuscript have the following competing interests: [insert competing interests here]*<br><br>* typeset	The authors have declared that no competing interests exist.
**Ethics Statement**<br><br>Enter an ethics statement for this submission. This statement is required if the study involved:<br><br>• Human participants<br>• Human specimens or tissue<br>• Vertebrate animals or cephalopods<br>• Vertebrate embryos or tissues	This study received ethical approval from the ICMR-NIRRCH Ethics Committee for Human Studies on November 13th, 2023 (D/ICEC/Sci-225/238/2023).<br>Written informed consent will be obtained from the parents of all participating children prior to their inclusion in the studyThis study received ethical approval from the ICMR-NIRRCH Ethics Committee for Human Bodies on November 13th, 2023 (D/ICEC/Sci-225/238/2023).<br>Written informed consent will be obtained from the parents of all participating children prior to their inclusion in the study

Powered by Editorial Manager® and ProduXion Manager® from Aries Systems Corporation

• Field research<br><br>Write "N/A" if the submission does not require an ethics statement.<br><br>General guidance is provided below. Consult the submission guidelines for detailed instructions. **Make sure that all information entered here is included in the Methods section of the manuscript.**	

Powered by Editorial Manager® and ProduXion Manager® from Aries Systems Corporation

**Format for specific study types**<br><br>**Human Subject Research (involving human participants and/or tissue)**<br>• Give the name of the institutional review board or ethics committee that approved the study<br>• Include the approval number and/or a statement indicating approval of this research<br>• Indicate the form of consent obtained (written/oral) or the reason that consent was not obtained (e.g. the data were analyzed anonymously)<br><br>**Animal Research (involving vertebrate animals, embryos or tissues)**<br>• Provide the name of the Institutional Animal Care and Use Committee (IACUC) or other relevant ethics board that reviewed the study protocol, and indicate whether they approved this research or granted a formal waiver of ethical approval<br>• Include an approval number if one was obtained<br>• If the study involved *non-human primates*, add *additional details* about animal welfare and steps taken to ameliorate suffering<br>• If anesthesia, euthanasia, or any kind of animal sacrifice is part of the study, include briefly which substances and/or methods were applied<br><br>**Field Research**<br><br>Include the following details if this study involves the collection of plant, animal, or other materials from a natural setting:<br>• Field permit number<br>• Name of the institution or relevant body that granted permission	
**Data Availability**<br><br>Authors are required to make the data underlying their research findings fully available, without restriction.<br><br>If the manuscript reports pilot or preliminary results, the data underlying those results must be made available at the time of publication. A Data Availability	N/A - No results are reported; N/A - No results are reported

Powered by Editorial Manager® and ProduXion Manager® from Aries Systems Corporation

Statement describing where the data can be found is required at submission. Your answers to this question constitute the Data Availability Statement and will be published in the article, if accepted. When legal or ethical requirements prohibit public data sharing, authors must indicate how researchers may obtain access to the data. See the PLOS Data Policy and FAQ for detailed information.<br><br>**Important:** Stating 'data available on request from the author' is not sufficient. If your data are only available upon request, select 'No' for the first question and explain your situation in the text box.<br><br>For protocols without pilot or preliminary data, authors are strongly encouraged to state how they plan to share research data from their study when it is completed or published.<br><br>If your study includes pilot data, will they be made fully available without restriction?	
**Describe where the data may be found in full sentences. If you are copying our sample text, replace any instances of XXX with the appropriate details.**<br><br>• If **no pilot data are reported** in the manuscript, please enter the following: *No datasets were generated or analysed during the current study. All relevant data from this study will be made available upon study completion.*<br>• If the manuscript reports pilot data **held in a public repository**, include URLs, accession numbers or DOIs. If this information will only be available after acceptance, indicate this by ticking the box below. For example: *All XXX files are available from the XXX database (accession number(s) XXX, XXX.).*<br>• If the manuscript reports pilot data contained **within the manuscript and/or**	Deidentified research data will be made publicly available when the study is completed and published.Deidentified research data will be made publicly available when the study is completed and published.

**Supporting Information files**, enter the following:<br>*All relevant data are within the manuscript and its Supporting Information files.*<br>• If neither of these applies but you are able to provide **details of access elsewhere**, with or without limitations, please do so. For example:<br><br>*Data cannot be shared publicly because of [XXX]. Data are available from the XXX Institutional Data Access / Ethics Committee (contact via XXX) for researchers who meet the criteria for access to confidential data.*<br><br>*The data underlying the results presented in the study are available from (include the name of the third party and contact information or URL).*<br>• This text is appropriate if the data are owned by a third party and authors do not have permission to share the data.<br><br>For study protocols with data management or sharing plans, authors are encouraged to briefly describe in their Data Availability Statement how data they generate will be made accessible when the study is completed. For example:<br>• Deidentified research data will be made publicly available when the study is completed and published.<br><br>* typeset	
Additional data availability information:	Tick here if the URLs/accession numbers/DOIs will be available only after acceptance of the manuscript for publication so that we can ensure their inclusion before publication.

Powered by Editorial Manager® and ProduXion Manager® from Aries Systems Corporation

Manuscript

Click here to access/download;Manuscript;IYCF PROTOCOL PAPER 2207.docx

Protocol for Study on Improving Infant and Young Child Feeding (IYCF) Practices in Tribal Block of Palghar District, Maharashtra through Involvement of Frontline Workers

Suchitra Surve[1], Shraddha Kyatam[2], Shahina Begum[4], Bhavya MK[4], Santosh Chaudhari[5], Sagar Patil[6], Shivshankar Timmanpyati[7], Purabi Mahajan[8], Ragini Kulkarni[9]*

[1]Scientist D, Department of Child Health Research, ICMR-NIRRCH Mumbai, Maharashtra, India

[2]Project Research Scientist-I (Non-medical), Department of Operational and Implementation Research, MRHRU Dahanu, ICMR-NIRRCH Mumbai, Maharashtra, India

[3]Scientist F, Department of Biostatistics, ICMR-NIRRCH Mumbai, Maharashtra, India

[4]Scientist B, Department of Operational and Implementation Research, Nodal Officer, MRHRU Dahanu, ICMR-NIRRCH Mumbai, Maharashtra, India

[5]District Health Officer, Office of the District Health Officer, District Headquarters Palghar, Maharashtra, India

[6]District RCH Officer, Office of the District Health Officer, District Headquarters Palghar, Maharashtra, India

[7]Chief Clinical Dietician, Tata Memorial Hospital, Mumbai, Maharashtra, India

[8]Senior Clinical Dietician, Tata Memorial Hospital, Mumbai, Maharashtra, India

[9]Department of Operational and Implementation Research, ICMR-NIRRCH Mumbai, Maharashtra, India

***Corresponding author: Ragini Kulkarni**

Dr. Ragini Kulkarni, Scientist F, Department of Operational and Implementation Research, Nodal Officer, MRHRU Dahanu, ICMR-NIRRCH Mumbai, Maharashtra, India, Email: kulkarnir@nirrch.res.in, kulkarnir120@gmail.com

Abstract:

Introduction: Infant and Young Child Feeding (IYCF) is crucial for child growth, especially in the first two years. Despite efforts, malnutrition remains high 35.5% of under-five children in India are stunted (2023). In Maharashtra, tribal areas like Palghar are most affected due to poverty, poor healthcare, and low awareness. Anganwadi Workers (AWWs) are key to IYCF promotion but face significant challenges. The study aims to assess the knowledge, attitude, and practices (KAP) of healthcare workers and mothers on IYCF. Based on the findings, an intervention package will be developed to address gaps and improve IYCF practices. The study will also compare children's nutritional status and key WHO IYCF indicators before and after the intervention, and document facilitating factors and challenges during its implementation.

Methods: A quasi-experimental study will be conducted over two years in three phases in the Ganjad PHC area in the tribal region of Palghar District, Maharashtra. Based on prior findings, 460 children will be enrolled each for the pre- and post-intervention surveys. Sampling will be done across 44 Anganwadi centers, with 10–11 eligible children selected per center. Infants aged 6–12 months, their mothers, and randomly selected ASHAs and AWWs will be included for In-depth interviews. Children with chronic illnesses, Severe Acute Malnutrition/Moderate Acute Malnutrition, birth defects, or those whose mothers do not provide written informed consent will be excluded.

Discussion: This study proposes a comprehensive, community-based intervention to improve IYCF practices among tribal populations in Palghar District, Maharashtra. It integrates behavior change communication with hot cooked meal provision at Anganwadi centers, leveraging frontline workers for implementation. The approach

emphasizes cultural sensitivity, sustainability, and local resource utilization. Findings may offer scalable solutions and inform policy within ICDS.

Clinical trial registration: CTRI/2024/06/068427 Registered on 06th June, 2024.

Keywords: Undernutrition, IYCF, complementary feeding, weaning, tribal children

1. Introduction:

Undernutrition remains a major contributor of child mortality, accounting for nearly 50% of child deaths. It mainly prevails during the initial two years of life which is a crucial window for child growth and development and when nutritional requirements are at the peak (1). It includes the commencement of complementary feeding which marks the transition from exclusive breast feeding or breast milk substitute to family foods, and entails gradual introduction of diverse foods gradually until the child adapts to eating from family pot (2), (3). It has a significant impact on nutritional status of a child. Inappropriate complementary feeding (CF) practices during this period are predominantly associated with growth faltering, malnutrition, micronutrient deficiencies, increased risk of infections and mortality during the first two years of life (4), (5), (6). Most common inappropriate CF practices are delayed introduction of complementary foods, inadequate nutritional content of weaning foods, low energy and nutrient density of foods offered, feeding in small amounts at meals, and food restrictions. It is influenced by interplay of various factors including cultural beliefs, poor hygiene, traditional feeding customs and local food availability in the region. Sub-optimal child feeding practices not only affect physical health of a child, but also impair cognitive and social development leading to poor academic performance. Therefore, it is critical to identify approaches to improve feeding practices particularly in the first two years of life to achieve optimal nutrition outcomes among children (7). Infant and Young Child Feeding (IYCF) practices consists of a set of widely accepted guidelines, recommended for appropriate feeding of new-born and children under two years of age (7).

WHO has identified eight main core indicators of IYCF practices namely- Early initiation of breastfeeding, exclusive breastfeeding for six months, continued

breastfeeding till completion of first year, Introduction of solid, semi-solid or soft foods, Minimum dietary diversity (MDD), Minimum meal frequency (MMF), Minimum acceptable diet (MAD), Consumption of iron rich or iron-fortified foods (3). However, these practices are often suboptimal in tribal regions due to poor nutrition, cultural beliefs, geographical disparities, poor socio-economic status and inadequate health facilities (8). This leads to undernutrition in tribal children as a result of inadequate nutritional intake and subsequent higher rates of morbidity (9). Frontline workers such as Accredited Social Health Activists (ASHAs), Anganwadi Workers (AWWs), and Auxiliary Nurse Midwives (ANMs) play a pivotal role in promoting behaviour change and providing nutrition education at the community level.

Several studies done so far in Indian population have shown that inappropriate IYCF practices are higher among tribal population (10), (11), (12). However, very few studies have evaluated impact of nutrition education on variety, quantity and consistency of CF. There is scarcity of data on assessing improvement in IYCF core indicators through community-based intervention (13), (14). Therefore, this study aims to improve IYCF practices in a tribal block of Palghar district through involvement of frontline workers for promoting better health outcomes among infants and young children.

2. Materials and Methods:

2.1. Study Setting

The study will be conducted in Primary Health Centre (PHC) area Ganjad which is located in the tribal region of Palghar District, Maharashtra, India (Fig 1). This area is predominantly inhabited by tribal communities (Kathodi, Katkari, Kokana, Koli

Mahadeo, Koli Malhar, Warli, Vanjari, Thakar, Thakur, Dubala, Dhor Koli, and Tokre Koli) and is characterized by limited access to healthcare services due to geographical disparities. The PHC serves as the primary healthcare facility for the surrounding villages (Table 1) and will act as the central site for implementing and monitoring the study activities.

Table 1. List of Villages with Number of Padas, Anganwadi Centers (AWCs), and Anganwadi Workers (AWWs) under Ganjad PHC

Village Name	Number of Padas	Number of AWCs	Number of AWWs
Ganjad	19	19	19
Raitali	7	7	7
Chari	11	11	11
Jamshet	15	15	15
Ambesari	12	12	12
Nagzari	13	13	13
**Total**	**77**	**77**	**77**

Abbreviations: AWC – Anganwadi Center, AWW- Anganwadi Worker, PHC- Primary Health Center

There are six subcentres in Ganjad PHC: Ganjad, Raitali, Chari, Jamshet, Ambesari, and Nagzari. The subcentre wise list of padas is mentioned in Table 1. Each pada is served by one Anganwadi Centre (AWC), resulting in 77 AWCs across the six villages. AWC is a community-based facility in India for children aged 0-6 years and pregnant or lactating women established under India's Integrated Child Development Services (ICDS) programme. It provides supplementary nutrition, immunization,

health check-ups, and preschool education to children less than six years of age, as well as aid to pregnant and breastfeeding mothers. Each center is managed by a trained Anganwadi Worker (AWW) and serves as an important link between the community and the health system, especially in rural and tribal areas, helping to reduce malnutrition, improve maternal and child health and support early childhood development. Correspondingly, there are 77 Anganwadi Workers (AWWs), with one assigned to each AWC and ten AWC are supervised by one AWC supervisor. Furthermore, Accredited Social Health Activists (ASHAs), a crucial part in rural health settings in India, are female community health workers, playing a key role in health awareness and community mobilization.

2.2. Study Population, Recruitment, and Sample Size

Considering 48.3% mothers who timely initiated complementary feeding at six months as per findings of previous study conducted by authors in Palghar District (15) and anticipating 10% improvement post intervention with 5% level of significance and 80% power, nearly 410 children will be required. Further, considering 10% non-response rate, 456 children will be enrolled. Therefore, an independent sample of 460 children will be selected in pre intervention and post intervention survey (10 to 11 children per AWC).

Additionally, as mentioned above, AWW, AWW Supervisor and ASHAs constituting frontline workers will also be study participants.

2.3. Study Framework

The study will be implemented over two years, in three phases, viz., Pre-intervention Phase, Intervention phase and post-intervention phase as depicted in study framework (Fig 2)

2.4. Pre-intervention Phase

Preparatory Activities

The protocol has been approved by Institutional Ethics Committee (D/ICEC/Sci-225/238/2023) and Clinical Trial Registration has been done (CTRI/2024/06/068427 Registered on 06th June, 2024).

During the pre-intervention phase, initially preparatory activities will include meetings with district health officials and training of staff recruited for the study. A comprehensive meeting with key district officials, including the Deputy Chief Executive Officer of the Women and Child Development Department, Palghar, and the Child Development Project Officer of Dahanu block. This meeting will serve to provide a detailed overview of proposed study, outlining its objectives, implementation strategies, and expected outcomes, thereby ensuring alignment and collaboration among all stakeholders to tackle malnutrition and promote optimal feeding practices.

Baseline data collection (Qualitative and quantitative)

As part of preparatory activities of the study, the situational analysis will be done from February 2024 to September 2024, through in-depth interviews (IDIs) with health care providers and frontline workers to collect information about their knowledge and perceptions about IYCF practices. The number of IDIs will be determined by convenient sampling. IDIs will be planned with CDPO, Anganwadi Supervisors, AWWs and ASHA workers. (Annexure 1.2, 1.3, 1.4, 1.5 - IDI Guide)

As part of the baseline data collection, information about IYCF practices and child's health status will be collected from 460 eligible mothers in a structured data collection form using the eight core indicators of IYCF practices. These include early initiation of breastfeeding, exclusive breastfeeding for the first six months, continued

breastfeeding at one-year, timely introduction of solid, MDD, MMF, MAD, full forms and the consumption of iron-rich or iron-fortified foods (Annexure 2). The inclusion criteria for the study consist of infants aged between 6 to 12 months who visit AWCs along with their mothers who are also enrolled in the study. The infants with chronic illnesses such as congenital heart disease, diabetes, cancer, or those diagnosed with Severe Acute Malnutrition (SAM), Moderate Acute Malnutrition (MAM), and birth defects like cleft lip or cleft palate will be excluded. Furthermore, any participant whose mothers does not provide consent will be excluded from the study.

The beneficiaries will be approached at Anganwadi Centres as shown in study framework. (Fig 2).

Additionally, a baseline survey will be conducted with ASHA workers and AWWs. They will be randomly selected through the Ganjad Primary Health Centre (PHC) area.

Preparation of Intervention Activities

To develop an intervention package, the existing IEC (Information, Education and Communication) materials on complementary feeding practices available at AWC will be reviewed, and improved IEC will be developed. A range of IEC materials will include a flip chart, video messages; pictorial charts etc. to promote behaviour change and improve IYCF practices. These will include information on optimal IYCF practices such as importance of exclusive breastfeeding, dos and don'ts of complementary feeding, continued breastfeeding and emphasis on iron and calcium-rich foods. Additional visual materials will feature information on appropriate semi-solid and soft foods, and key IYCF indicators such as MDD, MMF, and MAD. Pictorial recipe booklets and cooking videos demonstrating easy, nutritious recipes

using locally available ingredients will be developed for better understanding and recall. IEC material will be developed in consultation with an expert committee, comprising of Nutritionists, Paediatricians in accordance to IYCF Guidelines of National Health Mission (NHM) (7), Manual on Dietary Guidelines for India, ICMR-National Institute of Nutrition (NIN) (16), (17), (18), (19) (Annexure 3).

Pre-intervention phase will also include capacity-building sessions for AWWs along with audio visual sessions for AWWs and ASHAs focused on IYCF concepts. These sessions will include demonstrations of recipes made from locally available ingredients and the use of amylase-rich flour, with a special focus on simple and nutritious recipes derived from Take Home Ration (THR) provided at the Anganwadi's. Counselling sessions will also emphasize the importance of avoiding processed foods.

The intervention will be pretested at the end of the preparatory phase to assess feasibility, acceptability, and effectiveness before full-scale implementation.

2.5. Intervention Phase

The intervention phase will be initiated in October 2024. Infants aged 6 to 12 months and children's who regularly visit to the anganwadi Centers, along with their mothers will be included during intervention phase regardless of their inclusion in preintervention phase to ensure intervention to all infants in desired age group in the study area. whereas infants suffering from chronic illnesses such as congenital heart disease, birth defects, diabetes, cancer, or other severe health conditions, those already diagnosed with Severe Acute Malnutrition (SAM), Moderate Acute Malnutrition (MAM) or those who are not willing to participate will also be excluded during the intervention phase.

During the intervention phase, several structured activities will be introduced to promote optimal IYCF practices in a defined timeline with emphasis on following four domains of intervention package i.e.

- Provision of training and capacity building of frontline workers and mothers on IYCF Practices through SBCC (Socio Behavioral Communication Change approach)
- Preparation of food charts, pictorial booklets
- Provision of servings of complementary feeds at Anganwadi Centres
- Iron, Calcium and Vitamin D supplementation in infants

As shown in Fig 3, intervention activities will be planned as per the villages over a stipulated time period of one year.

Provision of training and Capacity building of frontline workers and Mothers on IYCF Practices (SBCC approach)

Training sessions for mothers will be conducted in groups at Anganwadi Centers. These sessions will focus on educating mothers about appropriate weaning foods, preparation of nutritious homemade recipes, feeding frequency, and age-appropriate portion sizes. Additionally, quarterly refresher training will be conducted for both AWWs and mothers to reinforce IYCF knowledge and practices throughout the intervention phase.

SBCC approach will predominantly constitute role plays, community activities, competitions, group sessions and crating role models in community through involvement of gatekeepers.

Preparation of food charts, pictorial booklets- AWW, MOTHERS

A nutritionist from the project team will provide weekly food preparation charts specifically designed for mothers of children aged 6–12 months. These charts will serve as practical guides for mothers.

In addition to the weekly charts, a range of IEC materials will be disseminated for AWWs and mothers to enhance understanding and encourage the adoption of recommended feeding practices. These materials will include illustrated cooking booklets with simple, nutritious recipes, food brochure that can be easily understood regardless of literacy levels. Live cooking demonstrations using locally available ingredients will be organized for mothers and short educational videos will be prepared for demonstrating food preparation techniques, outlining balanced meal plans with emphasis on avoiding processed foods.

To further support learning, live cooking demonstrations will be organized regularly at Anganwadi centres. These sessions will provide hands-on experience to mothers, showing them how to prepare nutritious meals using affordable, seasonal, and locally sourced ingredients. Such demonstrations will also be interactive, allowing mothers to ask questions and clarify doubts directly with the nutritionist.

Moreover, mothers will be educated on the importance of avoiding processed and packaged foods that are high in salt, sugar, and unhealthy fats. They will be encouraged to prepare fresh, home-cooked meals for their children and guided on recognizing unhealthy food options.

An essential component of this initiative will be the proper maintenance of diet cards. Each mother will be provided with a diet card to record daily feeding details, including the types of food given, quantity and frequency. (Annexure 4). The nutritionist and field staff will regularly review these cards to monitor progress,

provide personalized feedback, and ensure adherence to recommended feeding practices.

Provision of servings of complementary feeds at Anganwadi Centres

Anganwadi Workers (AWWs) will be responsible for preparing age-appropriate solid and semi-solid weaning foods at the Anganwadi Centers, ensuring the provision of hot cooked meals for children aged 6–12 months. Mothers will be encouraged to take two to three servings from the center for their children, while the remaining feeds for the day will be prepared at home. To support this, AWWs will receive provisions of ration supplies for food preparation by the research team.

Each mother will record daily feeding practices in the diet cards provided which will be monitored weekly by research team to track adherence and provide feedback. Mothers demonstrating good compliance and improvement in child feeding may be recognized through prizes and appreciation certificates for AWWs to encourage continued participation.

To facilitate communication and ongoing support, WhatsApp groups will be formed with AWWs, and project staff. These groups will be used to share reminders, tips, IEC materials, share photos of recipes and respond to queries, fostering a supportive environment for improving IYCF practices.

Monthly examinations of participating infants

To ensure child health and monitor impact, clinical examination will be carried out by a Medical Officer. These check-ups will include assessments for gastrointestinal and respiratory infections, as well as evaluations of growth patterns through weight and height measurements to determine nutritional status. Children will be screened for MAM, SAM, stunting, and underweight conditions using WHO growth

standards. Identified cases of SAM and MAM will be treated and referred appropriately.

Process Indicators (Weekly Monitoring)

As shown in Table 2, for ongoing monitoring, weekly process indicators will be recorded, including the number of children receiving at least two complementary feeds at the Anganwadi, number of mothers providing feeds at home (especially on holidays), and those availing feeds from AWCs. Data will also be collected on infants achieving MDD, MMF, and MAD, as well as the preparation and use of amylase-rich flour at AWCs. The use of food charts and recipe demonstrations by AWWs, the number of infants consuming iron-rich foods, and those receiving iron, calcium, and vitamin D supplements will also be tracked. Challenges and gaps identified during the process, along with corrective actions taken, will be documented thoroughly.

All this information will be maintained in a dedicated register managed by the research team, and health cards for each child will be prepared and updated regularly to support continuous monitoring and evaluation.

Table 2. Input, Process Indicators, Outcomes and Impact

Input	Process	Process Indicators	Outcome Indicators	Impact
➢ Resources<br>➢ Training<br>➢ IEC Material<br>➢ Replacing	➢ Training to AWW/ASHA/Mother<br>➢ Demonstration cooking once in a	➢ No. of AWW/ANM trained<br>➢ No. of mothers trained<br>➢ no. of demo sessions	➢ Increased in awareness among health care providers	➢ % of infants SAM and MAM, stunting, undernutrition<br>➢ availability of

strategy of THR to infants in age group of 6-12 months with hot cooked meal	week for mother<br>➢ Distribution of charts, booklets and videos<br>➢ Iron, calcium and vitamin D supplementation<br>Appropriate and timely weaning at Anganwadi's	conducted by AWW<br>➢ No. of Mothers providing complementary feeds at home on holidays<br>➢ No. of Mothers availing complementary feeds at Anganwadi's<br>➢ No. of Anganwadi's preparing Amylase rich flour at Anganwadi's<br>➢ No. of AWW using food charts, demonstration of preparation of recipes from locally available foods at least monthly for training mothers.<br>➢ Number of AWW preparing hot	and mothers<br>➢ % of infants who received continued breastfeeding at 1 year<br>➢ % of infants received solid, semi-solid /soft foods<br>➢ % of infants achieved MDD, MMF, MAD<br>➢ Consumption of iron rich foods<br>➢ % of	IEC material sustainability of intervention in program referral system

		cooked food instead of THR for complementary feeding in (6 -12 months children)<br>➢ Number of eligible children in age group 0-12 months who are monitored for growth once in a month.	infants with Improvement in growth parameters, incidence of infections<br>➢ % of infants received iron, calcium and vitamin D supplements	

Abbreviations: THR- Take Home Ration, AWW- Anganwadi Worker, ASHA- Accredited Social Health Activist, ANM- Auxiliary Nurse Midwife, MDD- Minimum Dietary Diversity, MMF- Minimum Meal Frequency, MAD- Minimum Acceptable Diet, SAM- Severe Acute Malnutrition, MAM- Moderate Acute Malnutrition

2.6. Post-Intervention Phase (Quantitative and Qualitative)

A post-intervention survey will be conducted for six months after the intervention phase to evaluate the impact of the intervention on IYCF practices. A total sample of 460 infants aged 6 to 12 months as per eligibility will be selected from the Ganjad PHC. These may not be same as included for the baseline survey as impact of the interventions in the study area will be assessed. A structured questionnaire as mentioned will be used to collect detailed information on feeding practices, guided by the eight core IYCF indicators. The post-intervention will also include qualitative data collection as done during pre-intervention phase among ASHAs and AWWs by convenient sampling to assess their knowledge and role in promoting appropriate feeding behaviors. The data collection along with the recruitment of participants is expected to be completed by January 2026.

The outcome indicators as shown in Table 2 will be assessed in the post intervention phase. The outcome indicators will specifically measure the percentage of infants who are exclusively breastfed, those who continue breastfeeding up to two years, and those who begin receiving complementary foods at six months. It will also assess the proportion of infants who meet the recommended standards for dietary diversity, meal frequency, and overall dietary adequacy. Additionally, the study will evaluate the percentage of infants consuming iron-rich foods and receiving essential micronutrient supplements like iron, calcium, and vitamin D. An important component of the outcome will also be the percentage of ASHAs and AWWs who demonstrate improved knowledge and awareness of optimal IYCF practices. These outcome measures will help determine the overall effectiveness of the intervention. The projected results are expected to be achieved by February 2026.

2.6. Expected Outcome

The expected outcome of the intervention is to achieve a measurable improvement in IYCF practices among the target population in enhancing child nutrition and caregiver practices in the community. The community-based interventions planned in this study will enhance skills of health care workers along with empowering mothers with knowledge and skills thereby leading to improvement in complementary feeding practices which may result in eventual reduction in prevalence of SAM, MAM, undernutrition in the area. This model can be scaled up in other blocks of Palghar district and also in other tribal areas of India.

3. Discussion:

The study proposes a comprehensive targeted community-based approach to improve IYCF practices among tribal populations in Palghar District, Maharashtra. The available evidence has highlighted the gaps between recommended and current IYCF practices, necessitating need of strengthening and upgrading skills of the existing healthcare staff by making them more resourceful to improve the nutritional status of the children in community (20), (21). However, the present study builds upon previous experience of authors in same tribal block demonstrating suboptimal IYCF practices and impact of multicomponent intervention on nutrition of under-five children (15). However, going a step ahead, the current study lays out specific strategies to enhance IYCF practices in tribal region of Palghar district.

While national initiatives like ICDS and POSHAN Abhiyan of India seek to promote child nutrition, implementation in tribal areas is frequently hampered by logistical difficulties, cultural traditions, low literacy, and a lack of community engagement. Despite significant initiatives by Government of India to improve the nutritional status of under-five children, a large proportion of them are still

malnourished and there is a need to improve their nutritional status through specific targeted interventions. Our study plans integrating SBCC strategy with provision of hot cooked meal at Anganwadi's through involvement of frontline workers to address multiple facets of potential implementation challenges.

Under the ICDS programme, children aged 3-6 years receive supplementary nutrition in the form of hot cooked meals while children under three years of age receive THR ('Take home ration'). However, studies have shown that cooked food is more effective strategy of providing supplementary nutrition than THR packets since it is more acceptable, consumed, and has an adequate amount of calories and protein (22). Notably, our proposed intervention of providing hot cooked meal at Anganwadi's is in accordance to recommendations highlighted from earlier experiences from some of the states and tribal regions of Maharashtra demonstrating better acceptance and utilization of fresh hot cooked food to children below the age of 3 years (23), (24). Moreover, the emphasis on locally available food, visual educational aids, live recipe demonstrations, and tracking feeding practices will develop a culturally sensitive and feasible framework from perspective of sustainability.

Furthermore, capacity building of AWWs and ASHAs, developing role models in community will enhance community engagement. Moreover, tribal populations in India are affected by a trio of primary concerns including cultural impediments, socioeconomic difficulties and inaccessibility to the health system due to geographical disparities. This comprehensive strategy highlights frontline workers as village ambassadors for improved IYCF practices which may eventually translate into better anthropometric outcomes of tribal children.

The study puts forward a scalable and replicable strategy by focussing on critical period of first two years (730 days) of life contributing to "Journey first 1000 days" of

life aligning with objectives of WHO-UNICEF global nutrition target to improve nutritional outcomes of infants (25).

Additionally, the intervention may prove as feasible and long-term sustainable approach if adapted within existing ICDS program through utilization of allotted rations, locally available foods and existing resources. However, future assessment of cost effectiveness of this proposed intervention with the existing intervention may provide better insights for integration in the program through engagement of local stakeholders.

This study will address the need for context-specific interventions to enhance IYCF practices in tribal regions of India. The proposed model encompassing behaviour change communication, hot cooked meal provision at Anganwadi centers and empowering community may offer a sustainable and expandable solution to improve nutrition in the first two years of life.

Importantly, by leveraging potential of frontline health care workers to enhance IYCF practices in community, the study can pave a path for transforming child nutrition in tribal blocks. Importantly, the findings may provide crucial evidence for policy improvements and targeted interventions within ICDS and POSHAN 2.0 frameworks.

4. Ethics and Dissemination:

Funding: The authors disclosed receipt of the following financial support for the research, authorship, and/or publication of this article: This work was supported by the ICMR-NIRRCH [PMABHIM/C10/31//2023-24].

Institutional Review Board Statement: This study received ethical approval from the ICMR-NIRRCH Ethics Committee for Human Bodies on November 13th, 2023 (D/ICEC/Sci-225/238/2023)

Informed Consent Statement: Written informed consent will be obtained from the parents of all participating children prior to their inclusion in the study. Considering that the participants are children from rural and tribal areas, they are considered a vulnerable population. Therefore, special care will be taken to ensure ethical standards are upheld. The parents will be provided with a Participant Information Sheet clearly outlining the purpose and objectives of the study in a language they understand. Participation will be entirely voluntary, and no form of coercion or undue influence will be used to encourage participation. For illiterate parents, they will be explained the PIS in the local language in the presence of witness, their thumb impression will be taken and signature of witness will be taken. The privacy and confidentiality of all participants will be strictly maintained throughout the study. Personal and health-related data will be anonymized and handled with care. All physical records will be stored securely under lock and key, while electronic data will be stored in password-protected systems accessible only to the Principal Investigator and authorized research staff.

The findings of the study will be disseminated through a multi-pronged strategy to ensure that all key stakeholders, including policy-makers, health professionals, and community workers, are informed and engaged.

Author Contributions:

SS: Writing – original draft, review & editing, Conceptualization, Methodology, Project administration; RK: Writing – review & editing, Conceptualization,

Methodology; SB: Conceptualization, Methodology; SK: Writing – original draft, review & editing, Formal Analysis; BK: Writing – review & editing;

Acknowledgement:

We are deeply grateful to Dr. Geetanjali Sachdeva, Director, ICMR-NIRRCH (RA/1850/03-2025), for her valuable guidance and support. Our heartfelt appreciation goes to all the frontline health workers Anganwadi Workers (AWWs), Accredited Social Health Activists (ASHAs), and Anganwadi Supervisors for their time and insights. We also thank the Child Development Project Officer (CDPO) and the local health authorities of Palghar district for their cooperation in facilitating the data collection process.

Conflict of interest: The authors declare that the research was conducted in the absence of any commercial or financial relationships that could be construed as a potential conflict of interest.

Generative AI statement: The authors declare that no Gen AI was used in the creation of this manuscript.

Abbreviations:

IYCF	Infant and Young Child Feeding Practices,
WHO	World Health Organization
MDD	Minimum Dietary Diversity
MMF	Minimum Meal Frequency
MAD	Minimum Acceptable Diet
SAM	Severe Acute Malnutrition

MAM	Moderate Acute Malnutrition
PHC	Primary Health Center
ANM	Auxiliary Nurse Midwife
THR	Take Home Ration

References:

1. Arikpo D, Edet ES, Chibuzor MT, Odey F, Caldwell DM. Educational interventions for improving primary caregiver complementary feeding practices for children aged 24 months and under. The Cochrane database of systematic reviews. 2018 May;5(5).

2. UNICEF. Indicators for assessing infant and young child feeding practices - UNICEF DATA [Internet]. [cited 2024 Nov 29]. Available from: https://data.unicef.org/resources/indicators-for-assessing-infant-and-young-child-feeding-practices/

3. World Health Organization. UNCF (UNICEF). Indicators for assessing infant and young child feeding practices: definitions and measurement methods [Internet]. [cited 2025 Feb 3]. Available from: https://www.who.int/publications/i/item/9789240018389

4. Black RE, Allen LH, Bhutta ZA, Caulfield LE, de Onis M, Ezzati M, et al. Maternal and child undernutrition: global and regional exposures and health consequences. Lancet (London, England) [Internet]. 2008 [cited 2024 Nov 29];371(9608):243–60. Available from: https://pubmed.ncbi.nlm.nih.gov/18207566/

5. Motarjemi Y, Kaferstein F, Moy G, Quevedo F. Contaminated weaning food: a major risk factor for diarrhoea and associated malnutrition. Bulletin of the World Health Organization [Internet]. 1993 [cited 2024 Nov 29];71(1):79. Available from: https://pmc.ncbi.nlm.nih.gov/articles/PMC2393433/

6. Burgess A. Undernutrition in Adults and Children: causes, consequences and what we can do. South Sudan Medical Journal. 2008;

7. Ministry of Health & Family Welfare. Guidelines for Enhancing Optimal Infant and Young Child Feeding Practices [Internet]. Vol. 11, National Health Mission,Government of India. 2013. Available from: https://nhm.gov.in/images/pdf/programmes/child-health/guidelines/Enhancing-optimal-IYCF-practices.pdf

8. Venkaiah K, Damayanti K, Nayak MU, Vijayaraghavan K. Diet and nutritional status of rural adolescents in India. European Journal of Clinical Nutrition [Internet]. 2002 Nov 1 [cited 2025 May 12];56(11):1119–25. Available from: https://pubmed.ncbi.nlm.nih.gov/12428178/

9. Barry CE, Boshoff HI, Dartois V, Dick T, Ehrt S, Flynn JA, et al. The spectrum of latent tuberculosis: rethinking the biology and intervention strategies. Nature reviews Microbiology [Internet]. 2009 [cited 2024 Nov 29];7(12):845–55. Available from: https://pubmed.ncbi.nlm.nih.gov/19855401/

10. Sharma AK, Naheed Baig V, Yadav AK, Bharadwaj AK, Singh R, Sharma A. Prevalence and Risk Factors for Stunting Among Tribal Under-Five Children At South-West. National Journal of Community Medicine [Internet]. 2016;7(6):461–7. Available from: www.njcmindia.org

11. Birdi TJ, Joshi S, Kotian S, Shah S. Possible causes of malnutrition in Melghat, a tribal region of Maharashtra, India. Global journal of health science [Internet]. 2014 Sep 1 [cited 2025 Feb 2];6(5):164–73. Available from: https://pubmed.ncbi.nlm.nih.gov/25168997/

12. Sinha R, Dua R, Bijalwan V, Rohatgi S, Kumar P. Determinants of Stunting, Wasting, and Underweight in Five High-Burden Pockets of Four Indian States. Indian journal of community medicine : official publication of Indian Association of Preventive & Social Medicine [Internet]. 2018 Oct 1 [cited 2025 Feb 2];43(4):279–83. Available from: https://pubmed.ncbi.nlm.nih.gov/30662180/

13. Sethi V, Kashyap S, Seth V. Effect of nutrition education of mothers on infant feeding practices. Indian Journal of Pediatrics [Internet]. 2003 Jun 1 [cited 2025 May 12];70(6):463–6. Available from: https://pubmed.ncbi.nlm.nih.gov/12921312/

14. Kilaru A, Griffiths PL, Ganapathy S, Shanti G. Community-based nutrition education for improving infant growth in rural Karnataka. Indian Pediatrics. 2005;42(5):425–32.

15. Surve S, Kulkarni R, Patil S, Sankhe L. Impact of intervention on nutritional status of under-fives in tribal blocks of Palghar District in Maharashtra, India. Indian Journal of Public Health [Internet]. 2022 Apr 1 [cited 2023 Feb 2];66(2):159. Available from: https://journals.lww.com/ijph/fulltext/2022/66020/impact_of_intervention_on_nutritional_status_of.13.aspx

16. ICMR - National Institute of Nutrition I. छह माह से छोटे बच्चों का पोषण [Internet]. [cited 2025 May 26]. Available from: https://www.nin.res.in/ebooks/Flipchart-IV.pdf

17. ICMR - National Institute of Nutrition I. Improving Health and Nutritional Status of vulnerable segment of population by implementing multi-component Health and Nutrition intervention as a sustainable model of intervention [Internet]. [cited 2025 May 26]. Available from: https://www.nin.res.in/ebooks/Flipchart-II.pdf

18. ICMR - National Institute of Nutrition I. 6 माह से 2 साल के बच्चों का पोषण [Internet]. [cited 2025 May 26]. Available from: https://www.nin.res.in/ebooks/Flipchart-III.pdf

19. ICMR - National Institute of Nutrition I. Dietary Guidelines for Indians-(HINDI) [Internet]. [cited 2025 May 26]. Available from: https://www.nin.res.in/ebooks/Dietary_Guidelines_for_Indians (HINDI) booklet - A4.pdf

20. Avula R, Oddo VM, Kadiyala S, Menon P. Scaling-up interventions to improve infant and young child feeding in India: What will it take? Maternal & Child Nutrition [Internet]. 2017 Oct 1 [cited 2025 May 12];13(Suppl 2):e12414. Available from: https://pmc.ncbi.nlm.nih.gov/articles/PMC6866129/

21. Padmanabhan PS, Mukherjee K. Infant and Young Child Feeding (IYCF): A Gap Analysis between Policy and Practice. Indian Journal of Nutrition [Internet]. 2017 [cited 2025 Feb 3];(open). Available from: www.opensciencepublications.com

22. Marathe S, Yakkundi Deepali, Shukla Abhay. Is ‘Take Home Ration’ Really Improving the Nutritional Status of Children? A Study of Supplementary Nutrition for Under3 Children in Four Districts of Maharashtra. Journal of Community Nutrition & Health [Internet]. 2015;4(1). Available from: https://www.researchgate.net/publication/303013496

23. Hot cooked meal through Akshaya Patra for AWC childrens | UT of Dadra and Nagar Haveli and Daman and Diu | India [Internet]. [cited 2025 May 12]. Available from: https://ddd.gov.in/scheme/hot-cooked-meal-through-akshaya-patra-for-awc-childrens/

24. Policy Changes | mahan-trust [Internet]. [cited 2025 May 12]. Available from:

https://www.mahantrust.org/policy-changes

25. Ministry of Health & Family Welfare, National Health Mission G of I. Journey of The First 1000 Days [Internet]. Rashtriya Bal Swasthya Karyakram. 2018. Available from: https://nhm.gov.in/images/pdf/programmes/RBSK/Resource_Documents/Journey_of_The_First_1000_Days.pdf

Supplementary Information:

S1 Fig Study site in Palghar District

S2 Fig The Study Framework

S3 Fig The Intervention Activities

S1 Annexure 1 IDI tool

S2 Annexure 2 Mother interview tool & CRF

S3 Annexure 3 Complementary feeding guidelines

S4 Annexure 4 Diet Card

Figure

Click here to access/download;Figure;S1_Fig 1 The Study site in Palghar District.jpg

Fig 1: Study site in Palghar District

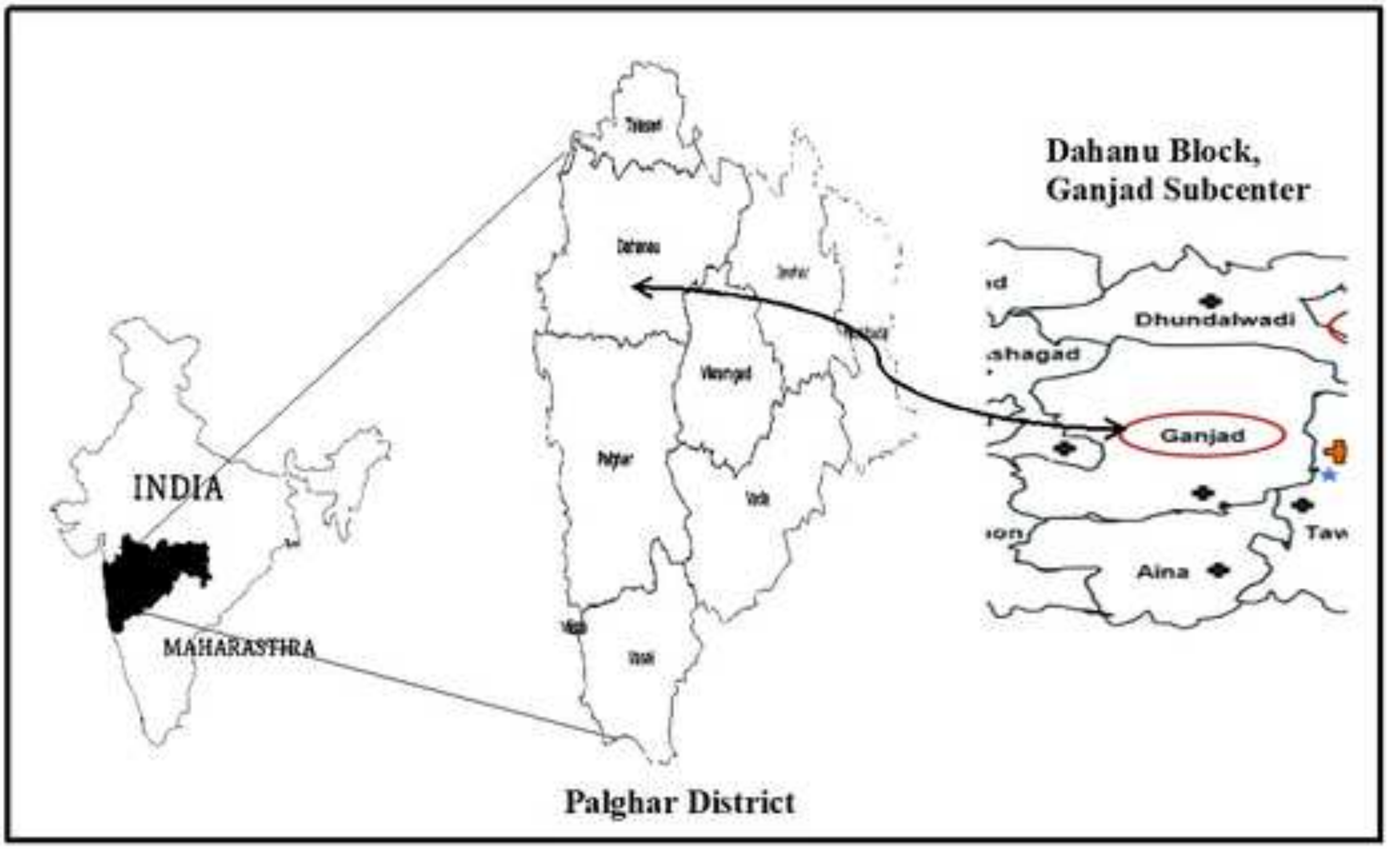

Legends: The map shows India highlighting Maharashtra, zoomed into Palghar district, marking Ganjad subcenter in Dahanu block

Figure

Click here to access/download;Figure;S2_Fig 2 The Study Framework.jpg

Fig 2: The study Framework

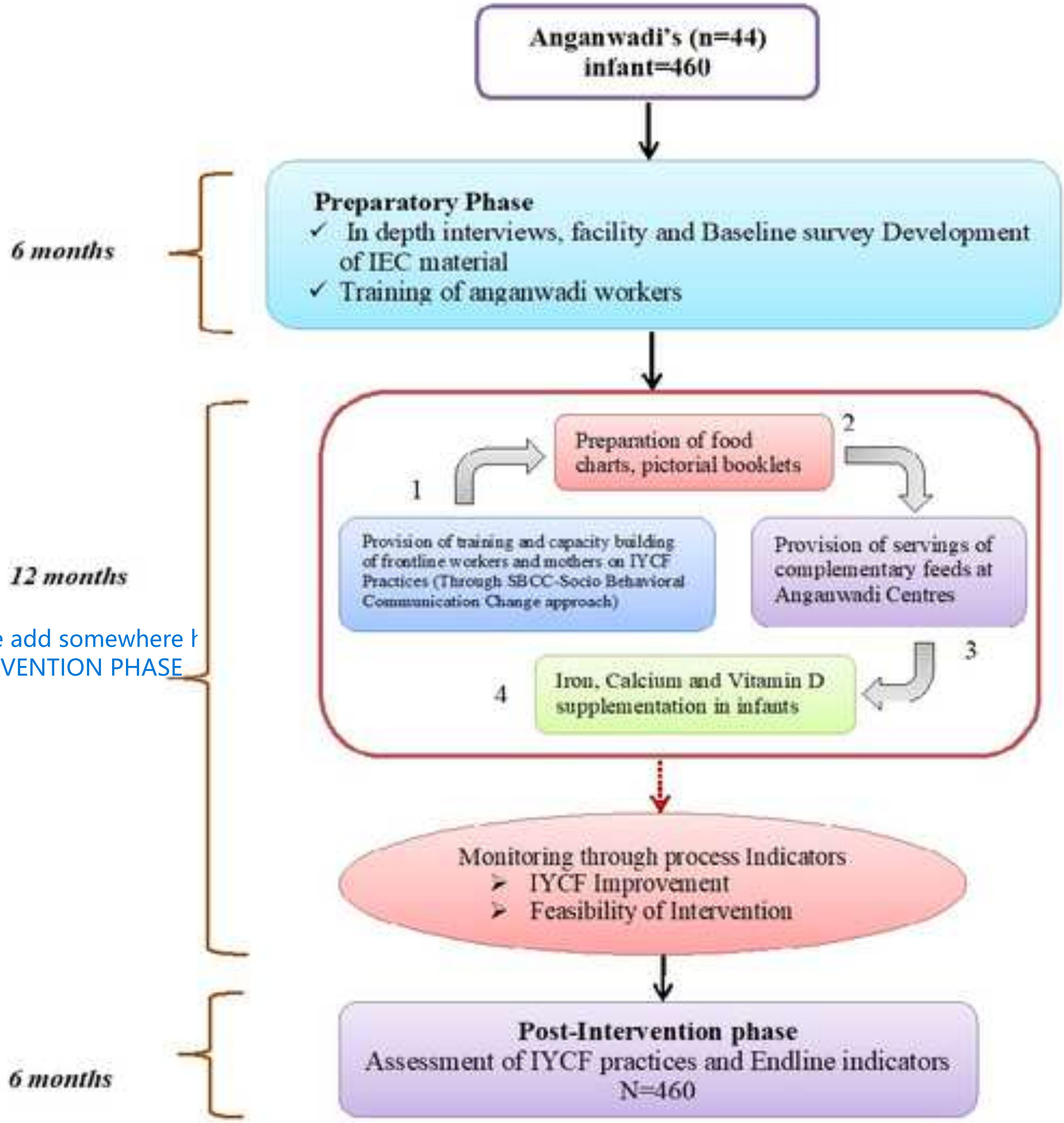

Please add somewhere h[illegible]
INTERVENTION PHASE

Abbreviation: PHC- Primary Health Center, AWW- Anganwadi Worker, ASHA- Accredited Social Health Activist, IYCF- Infant and Young Child Feeding Practices

Legends: The flowchart illustrates a phased intervention model at PHC-Ganjad involving 6 months preparatory activities, a 12-month intervention package, process monitoring, and post-intervention assessment across 44 Anganwadi's covering 460 infants.

Fig 3: The Intervention Activities

SR NO.	AWC / MONTH	Month 1	Month 2	Month 3	Month 4	Month 5	Month 6	SR NO.	AWC / MONTH	Month 7	Month 8	Month 9	Month 10	Month 11	Month 12
1	Masu 1, Masu 2, Masu Khalatpada	▲	●	■	✦	◆	⧗	15	A. Patilpada, Navapada, Vilhatpada, Koshipada, Bondpada	▲	●	■	✦	◆	⧗
2	Kakadpada, Sawarpada, Parawadi paraspada, Udhanipada	●	■	▲	✦	◆	⧗	16	Ubhadak pada, Kehlai pada, Chari Meghdevpada	●	■	▲	✦	◆	⧗
3	Ambesari Baripada, Jamshet Musalpada	■	▲	●	✦	◆	⧗	17	N. Bhonarpada 1, 2	■	▲	●	✦	◆	⧗
4	Ganjad Dabhipada, Vangadpada, Kalambipada	▲	●	■	✦	◆	⧗	18	Jamshet Kandipada 1, 2	▲	●	■	✦	◆	⧗
5	Ganjad Kandolpada, G Lakhanpada,	●	■	▲	✦	◆	⧗	19	Ambesari Materpada, Moraghpada, Khadkipada, kalangadpada	●	■	▲	✦	◆	⧗
6	Galanpada, Gujarpada, Kohralipada, Thakarpada, dongaripada	■	▲	●	✦	◆	⧗	20	C. Patil pada, Paras pada	■	▲	●	✦	◆	⧗
7	Raitali Patilpada, Chimbarpada, Kathepada, Dongaripada	▲	●	■	✦	◆	⧗	21	Jamshet Patilpada, Dongripada, Mangatpada, Kathipada	▲	●	■	✦	◆	⧗
8	Dasarapada, Diwanpada 1, 2, Somanpada	●	■	▲	✦	◆	⧗	22	J Khindipada, Shendepada	●	■	▲	✦	◆	⧗
9	Mothi khadaki, Lahan Khadkipada	■	▲	●	✦	◆	⧗	23	Nagzari Namberpada 1, 2	■	▲	●	✦	◆	⧗
10	Raitali Dalvipada, Ghadagpada	▲	●	■	✦	◆	⧗	24	Jamshet Tandelpada 1, 2	▲	●	■	✦	◆	⧗
11	Chari Kotbiswarpada, bhonarpada	●	■	▲	✦	◆	⧗	25	Chari Waghatpada, Bijadpada	●	■	▲	✦	◆	⧗
12	Chari Kotbikutadpada, Chari Khindipada	■	▲	●	✦	◆	⧗	26	Ganjad Dandanpada, dhakpada	■	▲	●	✦	◆	⧗
13	N. Dongripada 1, 2, Varchapada	▲	●	■	✦	◆	⧗	27	Ganjad Sambarpada	▲	●	■	✦	◆	⧗
14	Tokarpada, Fatherpada	●	■	▲	✦	◆	⧗	28	Raitali Bhonarpada	●	■	▲	✦	◆	⧗

▲ PICTORIAL CHART & VIDEO DISPLAY

● ROLE PLAY

■ RECIPE MAKING

✦ Q & A SESSION/Training cum Group sessions

◆ COMPETITIONS FOLLOWED BY AWARD/PRIZE

⧗ NUTRITIONAL ASSESSMENT

Abbreviation: AWC- Anganwadi Center

Legends: The table displays the monthly schedule of IYCF activities across Anganwadi Centers, using symbols to represent pictorial/video displays, role plays, recipe making, group sessions, competitions, and nutritional assessments.

Click here to access/download
Supporting Information
S1_Annexure 1 IDI tool.docx

Click here to access/download
Supporting Information
S2_Annexure 2 Mother interview tool & CRF.docx

Click here to access/download
Supporting Information
S3_Annexure 3 Complementary feeding guidelines.docx

Click here to access/download
Supporting Information
S4_Annexure 4 Diet Card.docx

1. Please complete all items on the Human Participants Research Checklist that are relevant for your submission, by following this link:

http://journals.plos.org/plosone/s/file?id=dc11/PLOSOne_Human_Subjects_Research_Checklist.docx (Contact us at plosone@plos.org if you cannot access the document.) There may be overlap between the checklist items and other queries listed below; please address any duplicated queries both in your response email and on the checklist itself. Upload the completed Human Participants Research Checklist as file type "Other" when you re-submit your manuscript. This document is for internal journal use only and will not be published if your article is accepted. The requested information will help us to assess whether your submission complies with PLOS ONE's policies and adheres to applicable reporting standards. Note that your manuscript may be rejected if you provide incomplete or inadequate responses to the checklist questions and that changing the 'Section/Category' of your article does not affect this requirement.

Response: Thank you for your guidance. We have reviewed the Human Participants Research Checklist available and completed all applicable sections relevant to our submission. The checklist has been uploaded under the file type "Other" as instructed.

2. We note that you have indicated that you received funding for your study. If you received peer reviewed external funding, your submission may be considered for expedited editorial assessment. To be considered for expedited review, please upload the Funding letter you received, and English translation, as an 'Other' file. Please also provide proof that the funder administered independent peer review of your protocol. Examples of evidence include: a link to a description of the funder's peer-review process; a letter from the funding agency confirming the independent peer review process; a list of the names and credentials of the peer reviewers (if available).

Please note that we will not consider the following types of funding for expedited peer review: Funding from an author's institution, or any other non-independent source; Funding from a commercial source; Funding that was not granted for this specific project (e.g. general PhD funding or fellowship).

If you are unable to provide proof of peer-reviewed external funding for your study, please note this in your cover letter to avoid delays in processing your manuscript. More information related to the relevant journal policies is available from:

[https://journals.plos.org/plosone/s/submission-guidelines#loc-study-protocols].

Response: Thank you for your note regarding funding and the possibility of expedited editorial assessment. We confirm that our study received external funding through a peer-reviewed grant from [PM-ABHIM Scheme]. We have uploaded the official funding letter as a file labeled "Other" in accordance with your instructions.

3. Please note that Study Protocols must relate to a research study that has not yet generated results. Studies that have completed either recruitment or data collection will not be considered. For further information, please see

https://journals.plos.org/plosone/s/submission-guidelines#loc-study-protocols. Please ensure that your Methods section reports the status and timeline of the study, including an estimate of when A) participant recruitment will be completed (if applicable), B) data collection will be completed and C) results are expected. If any of these stages have already been completed, this should be indicated in the Methods section.

Response: Thank you for your guidance regarding the submission of Study Protocols. We confirm that this manuscript outlines a research study that has not yet produced results. In accordance with PLOS ONE's policies, we have revised the Methods section to clearly indicate the current status and timeline of the study. Specifically, we have added the statement: "*As part of preparatory activities of the study, the situational analysis will be done from February 2024 to September 2024*" (Page no- 9, Line no- 206–207), "*The intervention phase will be initiated in October 2024*". (Page no- 11, Line no- 256); "*The data collection along with the recruitment of participants is expected to be completed by January 2026"* (Page no-18, Line no- 370-371). "*The projected results are expected to be achieved by February 2026"* (Page no-18, Line no- 383)

We have also explicitly stated that no recruitment or data collection has been completed at the time of submission.

4. We note you did not include a copy of the original, (signed or stamped) dated IRB/research ethics committee approval letter or waiver for all study sites as an 'Other' file. In keeping with our submission guidelines, please upload the approval letter or waiver as an 'Other' file. More information related to the relevant journal policies is available from: [https://journals.plos.org/plosone/s/submission-guidelines#loc-guidelines-for-specific-study-types].

Response: Thank you for your comment. We acknowledge the requirement and apologize for the oversight. We have uploaded the original, dated IRB/research ethics committee approval letter as an 'Other' file in accordance with the submission guidelines.

PONE-D-25-38092
Protocol for Study on Improving Infant and Young Child Feeding (IYCF) Practices in Tribal Block of Palghar District, Maharashtra through Involvement of Frontline Workers

1. During our evaluation of the documents provided, we noted that your ethics approval letter did not cover the entire date range for participant recruitment and data collection. Before we can proceed further with the submission, please provide the ethics approval extension document(s) for the study. If the document is in another language, please also provide an English translation. Please note that if this document is not included when your manuscript is resubmitted, it may be rejected.

   Response: Thank you for your guidance. We have uploaded the Continuation letter of Ethics Committee as suggested
