# Supplementary material for: Protocol for a quasi experimental mixed method study on impact of intervention for improving Infant and Young Child Feeding (IYCF) practices in tribal block of Palghar District, Maharashtra, India through involvement of frontline workers
Source: PLoS One. 2026 Jul 15;21(7):e0353241. doi: 10.1371/journal.pone.0353241 (PMC13372156; doi:10.1371/journal.pone.0353241)
Supplement: Attachment — Submitted filename: PLOS One Comments.docx Rev 2.docx [file pone.0353241.s007.docx]

PONE-D-25-38092
Protocol for Study on Improving Infant and Young Child Feeding (IYCF) Practices in Tribal Block of Palghar District, Maharashtra through Involvement of Frontline Workers

1. During our evaluation of the documents provided, we noted that your ethics approval letter did not cover the entire date range for participant recruitment and data collection. Before we can proceed further with the submission, please provide the ethics approval extension document(s) for the study. If the document is in another language, please also provide an English translation. Please note that if this document is not included when your manuscript is resubmitted, it may be rejected.

**Response:** Thank you for your guidance. We have uploaded the Continuation letter of Ethics Committee as suggested
