# Supplementary material for: Protocol for a quasi experimental mixed method study on impact of intervention for improving Infant and Young Child Feeding (IYCF) practices in tribal block of Palghar District, Maharashtra, India through involvement of frontline workers
Source: PLoS One. 2026 Jul 15;21(7):e0353241. doi: 10.1371/journal.pone.0353241 (PMC13372156; doi:10.1371/journal.pone.0353241)
Supplement: Attachment — Submitted filename: Response to Reviewers Revised 0605.docx [file pone.0353241.s009.docx]

6^th^ May, 2026

To,
The Editor
PLOS ONE

Subject: Submission of Revised Manuscript and Response to Reviewer Comments

Respected Sir/Madam,

We sincerely thank you and the esteemed reviewers for the thorough evaluation of our manuscript. We are grateful for the insightful comments and suggestions by reviewers to improve the quality and clarity of the manuscript.

We have revised the manuscript in accordance with the reviewer’s suggestions. The title of the manuscript is revised as per suggestions as **“Protocol for a Quasi Experimental Mixed Method Study on Impact of Intervention for Improving Infant and Young Child Feeding (IYCF) Practices in Tribal Block of Palghar District, Maharashtra, India through Involvement of Frontline Workers”.**

We have carefully considered the reviewer’s comments and have made the necessary modifications in the manuscript. A detailed point-by-point response with corresponding page numbers to each comment is enclosed along with the revised version of the manuscript.

Thanking you

Sincerely,

Corresponding author

**Dr. Ragini Kulkarni**

MD Community Medicine

Scientist F, Department of Operational and Implementation Research

ICMR- National Institute for Research on Women’s Health (NIRWoH) (Formerly ICMR-NIRRCH)

E-mail: [kulkarnir120@gmail.com, kulkarnir@nirrh.res.in](mailto:kulkarnir120@gmail.com,%20kulkarnir@nirrh.res.in)

1. **Please ensure that your manuscript meets PLOS ONE's style requirements, including those for file naming. The PLOS ONE style templates can be found at**

**https://journals.plos.org/plosone/s/file?id=wjVg/PLOSOne_formatting_sample_main_body.pdf and**

[**https://journals.plos.org/plosone/s/file?id=ba62/PLOSOne_formatting_sample_title_authors_affiliations.pdf**](https://journals.plos.org/plosone/s/file?id=ba62/PLOSOne_formatting_sample_title_authors_affiliations.pdf)

**Response:** Thank you for your comment. We have carefully reviewed the PLOS ONE style requirements and ensured that our manuscript complies with all relevant guidelines, including file naming conventions. The manuscript has been formatted according to the PLOS ONE style templates.

2. **Thank you for stating the following in the Acknowledgements Section of your manuscript:**

**“The authors disclosed receipt of the following financial support for the research, authorship, and/or publication of this article: This work was supported by the ICMR-NIRRCH [PMABHIM/C10/31//2023-24].”**

**We note that you have provided funding information that is not currently declared in your Funding Statement. However, funding information should not appear in the Acknowledgements section or other areas of your manuscript. We will only publish funding information present in the Funding Statement section of the online submission form.**

**Please remove any funding-related text from the manuscript and let us know how you would like to update your Funding Statement. Currently, your Funding Statement reads as follows:**

**“Initials of the authors: SS Grant number: (CTU/PM ABHIM/17/10/46/2023-ECD-II). Full Name of Funder: Indian Council of Medical Research (ICMR). The funders did not play any role in the study design, data collection and analysis, decision to publish, or preparation of the manuscript”**

**Please include your amended statements within your cover letter; we will change the online submission form on your behalf.**

**Response:** Thank you for your observation. We have removed the funding-related information from the Acknowledgements section of the manuscript, in accordance with PLOS ONE guidelines.

We would like to update the Funding Statement as follows:

“SS: (CTU/PM ABHIM/17/10/46/2023-ECD-II). Indian Council of Medical Research (ICMR). The funders did not play any role in the study design, data collection and analysis, decision to publish, or preparation of the manuscript.”

3. **PLOS requires an ORCID iD for the corresponding author in Editorial Manager on papers submitted after December 6th, 2016. Please ensure that you have an ORCID iD and that it is validated in Editorial Manager. To do this, go to ‘Update my Information’ (in the upper left-hand corner of the main menu), and click on the Fetch/Validate link next to the ORCID field. This will take you to the ORCID site and allow you to create a new iD or authenticate a pre-existing iD in Editorial Manager.**

**Response:** We confirm that the corresponding author has an ORCID iD (0000-0001-6238-2436). However, it could not be successfully validated in the editorial manager system. The following warning message is being displayed after we attempted,

*“Warning: An identical, validated ORCID already exists in the database. An ORCID may be linked to only one record in the database; therefore, it is possible that another user record was previously created and the ORCID was validated under that account.”*

The ORCID iD of the corresponding author is: 0000-0001-6238-2436. We kindly request you to do the necessary changes in the system if possible.

4. **Your ethics statement should only appear in the Methods section of your manuscript. If your ethics statement is written in any section besides the Methods, please delete it from any other section.**

**Response**: Thank you for your comment. We have reviewed the manuscript and ensured that the ethics statement appears only in the Methods section. Any duplicate or misplaced instances in other sections have been removed in accordance with the journal’s guidelines.

Page no.: 178-195

5. **Please include a separate caption for each figure in your manuscript.**

**Response:** Thank you for your comment. We have revised the manuscript to include separate captions for each figure, in accordance with the journal’s guidelines.

Captions are as follows:

“Fig 1. Study site in Palghar District

The map shows India, highlighting Maharashtra, zoomed into Palghar district, marking Ganjad Primary Health Center (PHC) in Dahanu block.

Fig 2. Gantt Chart

Gantt chart illustrating the timeline of study phases and key activities over the two-year study period, including pre-intervention, intervention and post-intervention phases.

Fig 3. The Study Framework

The flowchart illustrates a phased intervention model at PHC-Ganjad involving 6 months preparatory activities, a 12-month intervention package, process monitoring and post intervention assessment across 44 Anganwadi’s covering 460 infants.

Fig 4. The Intervention Activities

The figure displays the monthly schedule of IYCF activities across Anganwadi Centers, using symbols to represent pictorial/video displays, role plays, recipe making, group sessions, competitions and nutritional assessments.”

Page no.: 130-135, 201-203, 207-210, 288-291.

6. **We note that Figure 1 in your submission contain map images which may be copyrighted. All PLOS content is published under the Creative Commons Attribution License (CC BY 4.0), which means that the manuscript, images, and Supporting Information files will be freely available online, and any third party is permitted to access, download, copy, distribute, and use these materials in any way, even commercially, with proper attribution. For these reasons, we cannot publish previously copyrighted maps or satellite images created using proprietary data, such as Google software (Google Maps, Street View, and Earth). For more information, see our copyright guidelines:** [**http://journals.plos.org/plosone/s/licenses-**](http://journals.plos.org/plosone/s/licenses-) **and-dcopyright.**

**We require you to either (1) present written permission from the copyright holder to publish these figures specifically under the CC BY 4.0 license, or (2) remove the figures from your submission:**

1. **You may seek permission from the original copyright holder of Figure(s) [#] to publish the content specifically under the CC BY 4.0 license.**

**We recommend that you contact the original copyright holder with the Content Permission Form** [**(http://journals.plos.org/plosone/s/file?id=7c09/content-permission-form.pdf)**](http://journals.plos.org/plosone/s/file?id=7c09/content-permission-form.pdf)) **and the following text:**

**“I request permission for the open-access journal PLOS ONE to publish XXX under the Creative Commons Attribution License (CCAL) CC BY 4.0** [**(http://creativecommons.org/licenses/by/4.0/).**](http://creativecommons.org/licenses/by/4.0/)) **Please be aware that this license allows unrestricted use and distribution, even commercially, by third parties. Please reply and provide explicit written permission to publish XXX under a CC BY license and complete the attached form.”**

**Please upload the completed Content Permission Form or other proof of granted permissions as an "Other" file with your submission.**

**In the figure caption of the copyrighted figure, please include the following text: “Reprinted from [ref] under a CC BY license, with permission from [name of publisher], original copyright [original copyright year].”**

2. **If you are unable to obtain permission from the original copyright holder to publish these figures under the CC BY 4.0 license or if the copyright holder’s requirements are incompatible with the CC BY 4.0 license, please either i) remove the figure or ii) supply a replacement figure that complies with the CC BY 4.0 license. Please check copyright information on all replacement figures and update the figure caption with source information. If applicable, please specify in the figure caption text when a figure is similar but not identical to the original image and is therefore for illustrative purposes only.**

**The following resources for replacing copyrighted map figures may be helpful:**

**USGS National Map Viewer (public domain):** [**http://viewer.nationalmap.gov/viewer/**](http://viewer.nationalmap.gov/viewer/)

**The Gateway to Astronaut Photography of Earth (public domain): W**[**http://eol.jsc.nasa.gov/sseop/clickmap/**](http://eol.jsc.nasa.gov/sseop/clickmap/)

**Maps at the CIA (public domain): https**[**://www.cia.gov/library/publications/the-world-factbook/index.html**](http://www.cia.gov/library/publications/the-world-factbook/index.html) **and https**[**://www.cia.gov/library/publications/cia-maps-publications/index.html**](http://www.cia.gov/library/publications/cia-maps-publications/index.html)

**NASA Earth Observatory (public domain):** [**http://earthobservatory.nasa.gov/**](http://earthobservatory.nasa.gov/)

**Landsat:** [**http://landsat.visibleearth.nasa.gov/**](http://landsat.visibleearth.nasa.gov/)

**USGS EROS (Earth Resources Observatory and Science (EROS) Center) (public domain):** [**http://eros.usgs.gov/#**](http://eros.usgs.gov/)

**Natural Earth (public domain):** [**http://www.naturalearthdata.com/**](http://www.naturalearthdata.com/)

**Response**: Thank you for this very useful suggestion. We have replaced the figure with a map that complies with the journal’s copyright requirements. The source of the map data has been clearly acknowledged, and the appropriate link has been provided in the figure caption in accordance with CC BY 4.0 guidelines.

Page no.: 133-135

7. **If the reviewer comments include a recommendation to cite specific previously published works, please review and evaluate these publications to determine whether they are relevant and should be cited. There is no requirement to cite these works unless the editor has indicated otherwise.**

Response: Thank you for your comment. The reviewers have not recommended any specific previously published works for citation. Therefore, no additional references have been included in response to this point.

**Additional Editor Comments:**

1. **The study type is missing from the title. It is mentioned that in-depth interviews will also be conducted. Both qualitative and quantitative components are present, as the author mentioned. Is it a mixed-method study?**

Response: Thank you for your comment. We acknowledge that both qualitative and quantitative components are included in the study, as in-depth interviews as well as structured data collection will be conducted. Accordingly, we have revised the title to clearly reflect that this is a mixed-methods study.

Page no.: 1-3

2. **The title of the study protocol does not align with the study objective.**

Response: Thank you for your comment. We have carefully reviewed the title in relation to the study objectives and revised it as

“*Protocol for a Quasi Experimental Mixed Method Study on Impact of Intervention for Improving Infant and Young Child Feeding (IYCF) Practices in Tribal Block of Palghar District, Maharashtra, India through Involvement of Frontline Workers*”

Page no.: 1-3

3. **What ethical issues may arise, and how will those be dealt with?**

**Response**: Thank you for your comment. As the participants in the present study will be mothers and children from rural and tribal areas, they are considered a vulnerable population. Therefore, mothers will be adequately informed about the purpose and objectives of the study through a participant information sheet (PIS), and written informed consent will be obtained prior to participation. Both PIS and informed consent will be in local language which will be understood by the participants. Participation will be entirely voluntary, and no coercion of any kind will be exercised on the mothers. For illiterate parents, they will be explained the PIS in the local language in the presence of witness, their thumb impression will be taken and signature of witness will be taken. Participants will be ensured that even if they refuse to participate in the study or withdraw from the study, they will continue to receive services from the government health facilities. Privacy and confidentiality will be strictly maintained throughout the study by ensuring anonymity and secure handling of data. All physical records will be stored under lock and key, while electronic data will be securely maintained in a password-protected computer or laptop of the Principal Investigator. Participants will also have the right to withdraw from the study at any point without any consequences, and any identified health issues during the study will be referred for appropriate care.

4. **The Gantt chart is missing in the protocol.**

**Response:** Thank you for your comment. We acknowledge that the Gantt chart was missing in the initial submission. It has now been added to the manuscript to clearly depict the study timeline and phases as “*Fig 2*”

Page no.: 201-203

5. **The data analysis plan is missing.**

**Response:** Thank you for your comment. We have now included a detailed data analysis plan in the Methods section of the manuscript, outlining the statistical and qualitative approaches to be used for analyzing the study data. Following details incorporated are as follows:

“Data Collection Tool and Statistical Analysis Plan

Data will be collected using a pre-tested structured questionnaire and recorded systematically. The collected data will be entered into Microsoft Excel and subsequently imported into Statistical Package for the Social Sciences (SPSS) software for analysis. Data cleaning and validation will be performed prior to analysis to ensure accuracy and completeness.

Descriptive statistics such as frequencies, percentages, means, and standard deviations will be used to summarize socio-demographic characteristics and key study variables. Inferential statistical tests (such as Chi-square test for categorical variables and t-test for continuous variables) will be applied to compare pre- and post-intervention outcomes. Regression analysis will be used to control for confounding factors. Statistical significance will be considered at p<0.05.

Qualitative data (Nvivo version) will be used for analysis of qualitative data. Data obtained from in-depth interviews will be transcribed verbatim and analyzed separately using 3thematic analysis. Codes and themes will be developed to identify key patterns, perceptions, and challenges related to IYCF practices and intervention implementation.”

Page no.: 401-415

6. **Which checklist will be followed up to enhance the quality of research?**

**Response:** Thank you for your comment. We will follow appropriate reporting guidelines to enhance the quality of the research. Checklists COREQ for qualitative research and TREND checklist for quasi- experimental study will be considered to ensure comprehensive and transparent reporting.

**Comments to the Author**

1. **Does the manuscript provide a valid rationale for the proposed study, with clearly identified and justified research questions?**

**The research question outlined is expected to address a valid academic problem or topic and contribute to the base of knowledge in the field.**

**Reviewer #1: Partly**

**Reviewer #2: Yes**

**Reviewer #3: Yes**

**Response:** We have refined the introduction as per suggestions to strengthen the rationale.

Page no.: 74-89

2. **Is the protocol technically sound and planned in a manner that will lead to a meaningful outcome and allow testing the stated hypotheses?**

**The manuscript should describe the methods in sufficient detail to prevent undisclosed flexibility in the experimental procedure or analysis pipeline, including sufficient outcome-neutral conditions (e.g. necessary controls, absence of floor or ceiling effects) to test the proposed hypotheses and a statistical power analysis where applicable. As there may be aspects of the methodology and analysis which can only be refined once the work is undertaken, authors should outline potential assumptions and explicitly describe what aspects of the proposed analyses, if any, are exploratory.**

**Reviewer #1: Partly**

**Reviewer #2: Yes**

**Reviewer #3: Yes**

**Response:** `Thank you for this important suggestion. We have now included methodological details about study design, sample size estimation and data analysis,

Page no.: 401-415.

3. **Is the methodology feasible and described in sufficient detail to allow the work to be replicable?**

**Descriptions of methods and materials in the protocol should be reported in sufficient detail for another researcher to reproduce all experiments and analyses. The protocol should describe the appropriate controls, sample size calculations, and replication needed to ensure that the data are robust and reproducible.**

**Reviewer #1: No**

**Reviewer #2: Yes**

**Reviewer #3: Yes**

**Response:** Thank you for this important suggestion. We have revised the methodological details about study design, sample size estimation and data analysis.

Page no.: 116-129, 155-177, 196-210

4. **Have the authors described where all data underlying the findings will be made available when the study is complete?**

**The** [**PLOS Data policy**](https://journals.plos.org/plosone/s/materials-and-software-sharing) **requires authors to make all data underlying the findings described in their manuscript fully available without restriction, with rare exception, at the time of publication. The data should be provided as part of the manuscript or its supporting information, or deposited to a public repository. For example, in addition to summary statistics, the data points behind means, medians and variance measures should be available. If there are restrictions on publicly sharing data**

**—e.g. participant privacy or use of data from a third party—those must be specified.**

**Reviewer #1: No**

**Reviewer #2: No**

**Reviewer #3: Yes**

**Response:** Thank you for your comment. We have now included a statement in the manuscript specifying where and how the data underlying the findings will be made available upon completion of the study, in accordance with journal guidelines. Following point has been added:

“Data Availability Statement

No datasets were generated or analysed during the current study. All relevant data from this study will be made available upon study completion. (As per other PLOS one articles)

Page no.: 420-422

5. **Is the manuscript presented in an intelligible fashion and written in standard English?**

**PLOS ONE does not copyedit accepted manuscripts, so the language in submitted articles must be clear, correct, and unambiguous. Any typographical or grammatical errors should be corrected at revision, so please note any specific errors here.**

**Reviewer #1: Yes**

**Reviewer #2: Yes**

**Reviewer #3: Yes**

**Response**: Thank you for your comment. We have reviewed it.

6. **Review Comments to the Author**

**Please use the space provided to explain your answers to the questions above and, if applicable, provide comments about issues authors must address before this protocol can be accepted for publication. You may also include additional comments for the author, including concerns about research or publication ethics.**

**You may also provide optional suggestions and comments to authors that they might find helpful in planning their study.**

**(Please upload your review as an attachment if it exceeds 20,000 characters)**

**Reviewer #1: The study is relevant for reducing and malnutrition and associated health loss. However, the abstract lacks tool, sampling methods, type of analysis and data collection tool. The introduction is very weak. The methods is incomplete.**

**E.g dose of intervention is lacking, type of analysis is lacking, impact is included and data quality assurance, baseline data is missed. Again, the title dectates practice where as the entire document describes about the perception, knowledge and practice.**

**Response:** Thank you for your valuable comments. We appreciate the reviewer’s observation and have revised the manuscript accordingly. The abstract has been strengthened to include details on the data collection tool, sampling methods, and type of analysis. The introduction has been expanded to provide a clearer rationale and context for the study. Additionally, the Methods section has been revised to include comprehensive details on the study design, intervention components, and data analysis plan to ensure completeness and clarity.

Page no: 28-55

**Reviewer #2: Research Summary and overall impression**

**This is an article detailing an intervention study that is being planned to address issues regarding Infant and Young Child Feeding (IYCF) in India. According to the authors, malnutrition remains high (~35.50%) despite efforts at combating it. Restricting themselves to an area in the Palghar District of Maharashtra, the team intends to carry out this study over a two-year period in three phases: (1) pre-intervention, which will involve in-depth interviews (IDIs) with local health workers and health promoters; interviews with 460 eligible mothers who would have agreed to participate in the study; as well as the preparations of logistics and materials needed for the intervention phase; (2) Intervention, which will involve the adequate equipping of health workers and promoters with logistics and materials to administer education and training to nursing mothers with regard to IYCF, as well as the provision of servings and food supplements to participants at health centers; and (3) post-Intervention, which will involve a survey conducted over six months to assess impact of intervention on mothers in selected area in the Palghar District.**

**There are a few observations which the authors would need to note:**

**Major Issue:**

**Line 176: Please provide more clarity on how the sample size of 460 was arrived at by indicating the formula which was used. No statistical formula was shown, only figures.**

**Also, what statistical tests would be conducted on the data collected both at the pre-intervention and post-intervention phases? Which statistical software(s) would be used? Would comparisons be conducted? If so, at what p-value would these comparisons be considered significant? P<0.05? p<0.01?**

**Minor Issues:**

**There are a number of issues with manuscript format and referencing formats (both in-text and in the References section). I have tried to point some out with in-text comments (please find attached article for your perusal). The authors should go through the article and ensure all formats (text, referencing, and image) adhere to PONE requirements.**

**Other/General Remarks:**

**I think the study was well-presented. Working on the above suggestions should greatly enhance the article. I would recommend it for publication.**

**Response:** Thank you for your comment. We have revised the manuscript to provide greater clarity on the sample size estimation by including the appropriate statistical formula and assumptions used to arrive at the sample size of 460 participants.

We have also detailed the statistical analysis plan, specifying that data will be analyzed using SPSS software. Appropriate statistical tests, including descriptive statistics and inferential tests such as the Chi-square test and t-test, will be used to compare pre- and post-intervention outcomes. Statistical significance will be considered at p<0.05.

Page no: 155-166

**Reviewer #3: It is a very interesting study regarding feeding practices and nutrition in the first two years of life. Further research is needed in order to reduce malnutrition and improve nutritional status of infants.**

Response: We thank the reviewer for highlighting the importance of research on infant feeding practices and nutrition. We agree that further evidence is needed to reduce malnutrition and improve infant nutritional outcomes, and our study aims to contribute to this area, particularly in a tribal setting. We also plan to conduct further larger studies to reduce malnutrition and improve nutritional status of infants.

7. **PLOS authors have the option to publish the peer review history of their article (**[**what does this mean?**](https://journals.plos.org/plosone/s/editorial-and-peer-review-process#loc-peer-review-history)**). If published, this will include your full peer review and any attached files.**

**If you choose “no”, your identity will remain anonymous but your review may still be made public.**

**Do you want your identity to be public for this peer review? For information about this choice, including consent withdrawal, please see our** [**Privacy Policy**](https://www.plos.org/privacy-policy)**.**

**Reviewer #1: No**

**Reviewer #2: No**

**Reviewer #3: No**

**[NOTE: If reviewer comments were submitted as an attachment file, they will be attached to this email and accessible via the submission site. Please log into your account, locate the manuscript record, and check for the action link "View Attachments". If this link does not appear, there are no attachment files.]**

**To ensure your figures meet our technical requirements, please review our figure guidelines: https://journals.plos.org/plosone/s/figures**

**You may also use PLOS’s free figure tool, NAAS, to help you prepare publication quality figures: https://journals.plos.org/plosone/s/figures#loc-tools-for-figure-preparation.**

**NAAS will assess whether your figures meet our technical requirements by comparing each figure against our figure specifications.**
